# The genetic landscape of origins of replication in *P. falciparum*

**DOI:** 10.1093/nar/gkad1103

**Published:** 2023-12-01

**Authors:** Casilda Muñoz Castellano, Laurent Lacroix, Emilie Mathis, Paulina Prorok, Magali Hennion, Jose-Juan Lopez-Rubio, Marcel Méchali, Ana Rita Gomes

**Affiliations:** LPHI, CNRS, Université de Montpellier, 34095 Montpellier, France; Institut de Biologie de l’Ecole Normale Supérieure (IBENS), Ecole Normale Supérieure, CNRS, INSERM, Paris, France; LPHI, CNRS, Université de Montpellier, 34095 Montpellier, France; Institute of Human Genetics, CNRS, 34396 Montpellier, France; Université Paris Cité, CNRS, Epigenetics and Cell Fate, F-75013 Paris, France; LPHI, CNRS, Université de Montpellier, 34095 Montpellier, France; Institute of Human Genetics, CNRS, 34396 Montpellier, France; LPHI, CNRS, Université de Montpellier, 34095 Montpellier, France

## Abstract

Various origin mapping approaches have enabled genome-wide identification of origins of replication (ORI) in model organisms, but only a few studies have focused on divergent organisms. By employing three complementary approaches we provide a high-resolution map of ORIs in *Plasmodium falciparum*, the deadliest human malaria parasite. We profiled the distribution of origin of recognition complex (ORC) binding sites by ChIP-seq of two *Pf*ORC subunits and mapped active ORIs using NFS and SNS-seq. We show that ORIs lack sequence specificity but are not randomly distributed, and group in clusters. Licensing is biased towards regions of higher GC content and associated with G-quadruplex forming sequences (G4FS). While strong transcription likely enhances firing, active origins are depleted from transcription start sites. Instead, most accumulate in transcriptionally active gene bodies. Single molecule analysis of nanopore reads containing multiple initiation events, which could have only come from individual nuclei, showed a relationship between the replication fork pace and the distance to the nearest origin. While some similarities were drawn with the canonic eukaryote model, the distribution of ORIs in *P. falciparum* is likely shaped by unique genomic features such as extreme AT-richness—a product of evolutionary pressure imposed by the parasitic lifestyle.

## Graphical Abstract

**Graphical Abstract:**
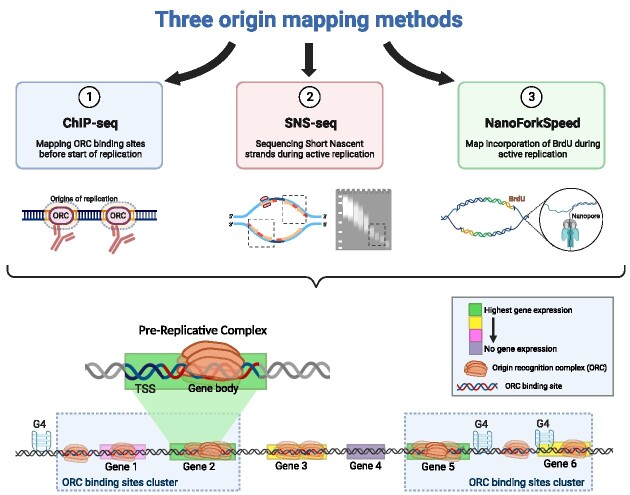

## Introduction

DNA replication is a highly orchestrated process that initiates from multiple sites in the genome. These sites are termed origins of replication (ORI). In model eukaryotes DNA replication is initiated during the G1 phase of the cell cycle, with the recruitment of the highly conserved six-subunit Origin Recognition Complex (ORC) to the ORIs. Subsequently, ORCs act as a landing platform for the assembly of the replisome, i.e. the replication machinery, in a process termed initiation. Initiation occurs in several steps: (i) recognition (ii) licensing of the origins, (iii) DNA unwinding and (iv) loading of the replisome including the DNA polymerase holoenyzmes and SSB (single-stranded DNA binding protein) (1,2). Specification of the ORI constitutes a crucial aspect of replication initiation. Origin's number, structure and usage dynamics are not conserved across organisms. With the exception of the budding yeast ORIs which harbour a 12-bp consensus sequence, ORIs are rarely defined by sequence elements (3–5). Instead, they are associated with features such as sequence composition, including AT-rich regions (6,7) and CpG islands (8–11); DNA topology, like G-quadruplexes (12–14), chromatin structure and status such as nucleosome-free chromatin (15); histone modifications (16–18); transcription (19,20), with some origins found at promoters (7,15,21).

**Figure 1.**
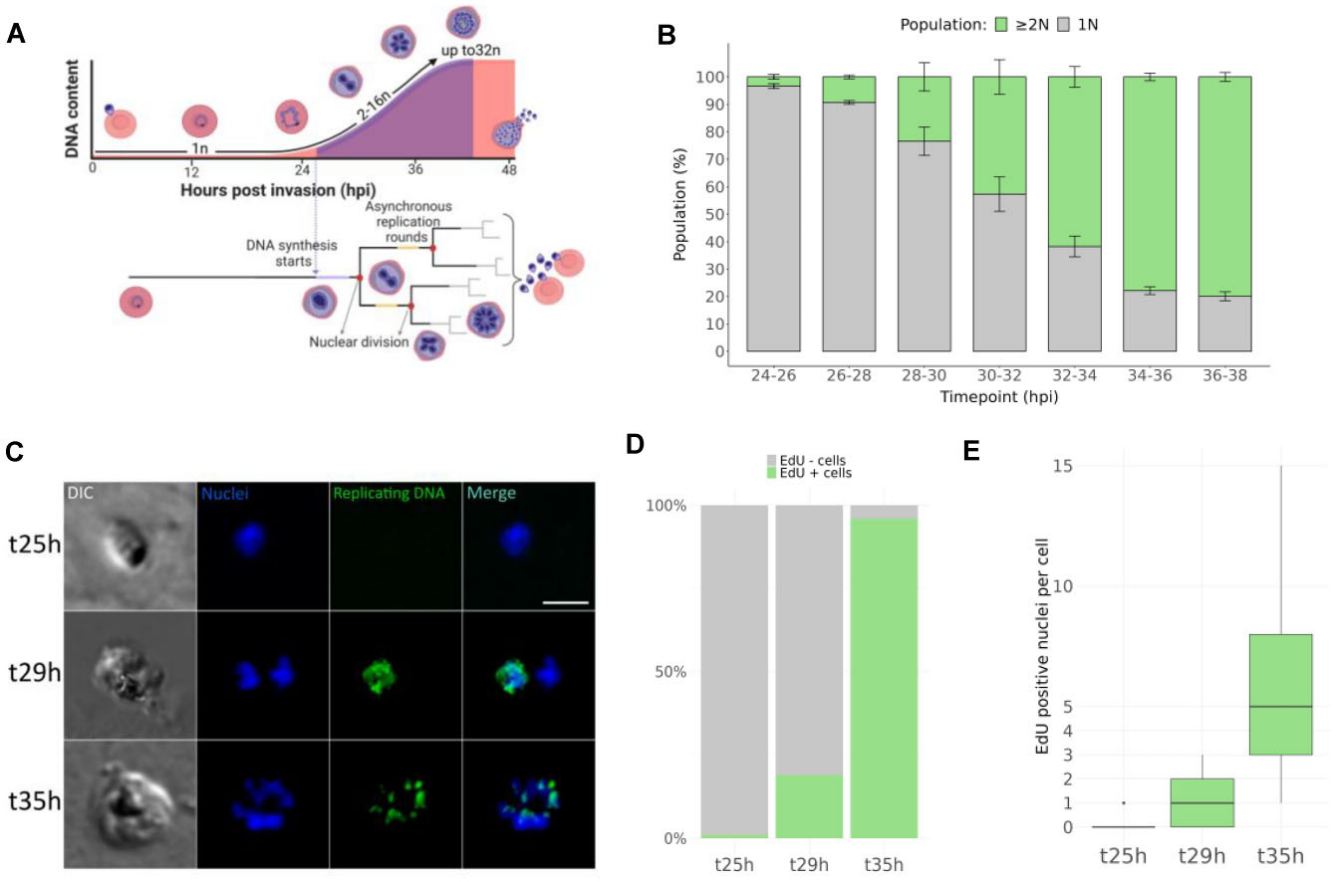
*Plasmodium falciparum* replication timing. (**A**) Schematics of the intra-erythrocytic *Plasmodium falciparum* asexual cycle highlighting DNA content increase in each cell and nuclear divisions over time. ’*n*’ denotes de number of nuclei in each parasite. Created with BioRender.com. (**B**) Total DNA content of *P. falciparum* (3D7) parasites measured by flow cytometry over time. Invasion window 2 h. hpi = hours post invasion. (**C**) Visualisation of active replication during schizogony after a 1 h incubation with 100 μM of EdU, followed by click-chemistry labelling with a fluorophore. Total DNA is stained with DAPI. Scale bar: 2 μm. (**D**) Percentage of parasites in which DNA replication took place (*i.e*. EdU positive) at various timepoints. At least one hundred parasites were counted for each timepoint. (**E**) Number of nuclei showing positive EdU signal per parasite. More than 40 parasites were counted for each timepoint.

DNA replication is an understudied topic in apicomplexan organisms. This group of divergent organisms includes various genera of pathogenic protozoa that cause diseases in humans and in a number of economically important animal species (22). In general, apicomplexan parasites have complex life cycles and employ, atypical multiplication strategies, which are tailored for fast population expansion during host colonisation and transmission. One example is the human malaria-causing parasite *Plasmodium falciparum*, responsible for the death of half a million children under the age of five, each year (23). The success and pathogenicity of *Plasmodium* is linked to its high multiplication rate (24). During vegetative growth within the human host, first in the liver (asymptomatic) and then in red blood cells (RBCs), malaria parasites expand their population by several orders of magnitude (25), and therefore interventions that impede this amplification have the potential to be particularly efficacious. Specifically, following invasion of the human RBCs, *P. falciparum* multiplies by schizogony, a process repeated every ∼48 h. This involves not one, but 4–5 cycles of asynchronous DNA replication rounds and karyokinesis, which take place prior to cytokinesis. This allows the production of 8 to 32 daughter cells in each cell cycle round, a surprisingly variable number that does not necessarily follow 2^*n*^ geometric expansion rules (26,27). Overall, DNA synthesis spans a period of ∼15 h (Figure 1A) (28,29), although each S-phase lasts on average <1 h (29). With multiple S phases in each intra-erythrocytic cycle, the identification of the DNA replication origins in the genome represents an important step towards understanding regulation of DNA replication in malaria parasites.

A variety of experimental approaches in a number of studies have been used to describe the nature and positioning of replication origins (30). However, individually, these methods such as marker frequency analysis sequence (MFA-seq (31,32)), DNA combing (33), short nascent strand enrichment (SNS-seq (10)), chromatin immunoprecipitation of ORC proteins followed by sequencing (ChIP-Seq (15)), among others (30), lack the necessary resolution to completely understand how origins are established.

Here we have combined three complementary approaches to obtain a robust cartography of the plasmodial origins of replication. Using ChIP-seq we report the first genome wide distribution of two elements of the ORC complex (*Pf*ORC1 and *Pf*ORC2) establishing a repertoire of putative licensed origins for *P. falciparum* parasites. Subsequently, we mapped sites of active replication using two strategies: (i) by sequencing DNA nascent strands (SNS-seq) at the onset of the first S-phase and (ii) by mapping the incorporation of the thymidine analogue BrdU into replicating DNA using nanopore sequencing, at two different times during schizogony (NFS).

Our combined datasets provide a comprehensive view of origin licensing and activation in the divergent malaria parasite *P. falciparum* and place this in the context of what is known in model eukaryotes.

## Materials and methods

### *P. falciparum* cell culture

Parasites (3D7) were cultured in O or A type human erythrocytes acquired from the Etablissement Français du sang (21PLER2018-0057) as a 5% suspension in RPMI 1640 medium (Gibco Life Technologies) supplemented with 25 mM HEPES, 50 mg/l hypoxanthine, 5% AB + human serum, 0.5% Albumax and 10 μg/ml gentamicin. Cultures were kept at 37°C in a controlled gaseous environment containing 5% O_2_, 5% CO_2_ and 90% N_2_. To achieve a semi-synchronous parasite culture, we used the Percoll/sorbitol method to first enrich the culture with parasites in the late stages of schizogony with a Percoll density gradient, followed by the selective elimination of the non-invaded parasites with 5% sorbitol after a 2 h re-invasion window. Parasite development was monitored on Giemsa stained thin-blood smears.

### Time-course of DNA replication

To determine the timing of DNA replication of our *P. falciparum* 3D7 lab strain, we measured the DNA-content of parasites by flow cytometry in a time-course fashion spanning the reported initiation timing (34). Briefly, following synchronisation with a 2 h invasion window as detailed above, parasites were harvested hourly from 24 h post-invasion (hpi) to 36 hpi and stained with VybrantTM DyeCycle™ Green (VG; 5 μM) (V35004, ThermoFisher Scientific) to stain the DNA of the parasites and MitoTracker™ Deep Red FM (MT; 1 μM) (M22426, ThermoFisher Scientific) to label viable mitochondrion according to their membrane potential. Fluorescence in each timepoint was measured in triplicate samples on the BD FACSAriaTM III (Becton Dickinson), using the red laser, 670/14 nM filter for MT and the green laser, 530/30 nM filter for VG. Data analysis was done using FlowJoTM v.10.8.1 software. A ring-stage sample and an asynchronous sample were used to gate parasites having one nucleus (gate 1N, i.e. non-replicating parasites) from those having two or more nuclei (gate ≥ 2N). These gates were applied to all timepoints.

### Parasite transfection

Parasites were transfected by electroporation of ∼5% ring-stage-infected RBCs with a total of 50–60 μg of circular plasmids as previously described (35). Electroporation was performed using a Bio-Rad Gene Pulser with settings 310 V, 950 μF and 200 Ω in 0.2 cm cuvettes. After electroporation, 100 μl of fresh RBCs were added to the suspension of parasites and these were grown under agitation, and selected with 2.5 nM of WR99210 (from Jacobus Pharmaceuticals; New Jersey, USA) 8–12 h after. The propagation of transgenic parasites was monitored by Giemsa-stained thin-blood smears. The *Pf*ORC HAx3 tagged lines *Pf*ORC1:HAx3 and *Pf*ORC2:HAx3 were selected with 2.5 nM of WR99210 (from Jacobus Pharmaceuticals; New Jersey, USA) for 12 days and subsequently grown without drug selection. Clones were isolated from these mutants by limiting dilution in the absence of drug pressure. For each transfection, both mixed population and clones were screened for correct gene edition and absence of WT locus by PCR. The line expressing the thymidine kinase episomally (TK^+^) was continuously selected with 2.5 nM of WR99210.

### Generation of plasmids

The genomic DNA used in all cloning reactions was extracted using the NucleoSpin Blood kit (Macherey Nagel). Before extraction, erythrocytes were lysed in a solution of 0.15% saponin (Sigma) in PBS, for 5 min on ice, and parasites were harvested by centrifugation (3000G, at 4°C, 5 min). To amplify each of the cloning fragments, KAPA HiFi polymerase (Roche) was used and the colony screening was done with GoTaq DNA Polymerase (Promega) following the manufacturer's instructions in both cases. gRNAs were ordered as oligonucleotides and annealed with a descending temperature gradient, phosphorylated with T4 PNK for 30 min at 37°C and cloned into the pDC2-Cas9-hDHFR-yFCU plasmid (36) after BbsI digestion using T4 DNA ligase (overnight at 16°C). All restriction enzymes, T4 DNA ligase and T4 PNK were purchased from NEB. Five different plasmids were used in this study:

*TK+*: A plasmid containing a previously described viral thymidine kinase was kindly provided by Catherine Merrick (37). We then used it to generate the TK + *P. falciparum* parasite line.

ORC1:HAx3: To introduce a triple haemagglutinin (HAx3) tag at the C terminus of *Pf*ORC1(PF3D7_1203000) via a double crossover homologous recombination event, two donor sequences were prepared to target the region immediately upstream (homology region 1; HR1) and downstream of the stop codon (homology region 2; HR2). The HR1 was assembled using two different fragments: one amplified using primers 90/91 (640 bp) from genomic DNA and a second fragment which was obtained as a G-block (IDT), spanning the last 186 bp of the gene but containing 6 shield mutations and a C-terminal triple haemagglutinin (HAx3) tag. The HR2 was amplified using primers 94/95 (845 bp) from genomic DNA. Both HRs were cloned sequentially into the plasmid pLN-HAx3 by In-Fusion HD cloning (Clontech) following digestion with AvrII and AflII (HR1) or AflII and BamHI (HR2). We selected a sgRNA targeting the 3′ end of the gene and the sgRNA sequence (annealed primers g19/g20) was cloned into the pDC2-Cas9-hDHFR-yFCU plasmid (36). Both plasmids were transfected simultaneously to obtain a scarless insertion of the tag. The resulting parasite line was named *Pf*ORC1::HA.

ORC2:HAx3: To introduce a HAx3 tag at the C terminus of *Pf*ORC2 (PF3D7_0705300), we used primers 86/87 (597 bp) to amplify HR1 and primers 88/89 (462 bp) to amplify HR2. Both HRs were amplified from genomic DNA and sequentially cloned into the plasmid pLN-HAx3 by In-Fusion HD cloning (Clontech) following digestion with AvrII and NaeI (HR1) or AflII and BamHI (HR2). The annealed primers g25/g26 were cloned into the pDC2-Cas9-hDHFR-yFCU plasmid (36) as described above. After recombination of the HAx3 tag into the genome, the targeting sgRNA sequence was split in two hence eliminating the need of incorporating shield mutations in the construct. The resulting parasite line was named *Pf*ORC2::HA.

A list of all primers that were used for genotyping, cloning and CRISPR–Cas9 guides is provided in Supplementary Table S4.

### Immunofluorescence assays

Active replication: Synchronised TK+ parasites were incubated with 100 μM 5-ethynyl-2′-deoxyuridine (EdU) for 1 h in standard culture conditions at either 25, 29 or 35 h post invasion (hpi). After, 200 μl of pelleted infected RBCs (∼4%) were spun down at 900 g and then washed once with 1% BSA in PBS. Pellets were then fixed with 4% paraformaldehyde for 15 min at room temperature and then washed three times with 1% BSA in PBS. Next, cells were permeabilised incubating in 0.01% Triton X-100 and 0.015% saponin in 1 ml of PBS for 15 min, at room temperature. After, cells were washed three times in 1 ml of 1% BSA in PBS. EdU signal was detected with click chemistry (Click-iT^®^ EdU flow cytometry assay kit from Molecular Probes, catalog number C10419). Parasite pellets were incubated in click reaction buffer (2 mM CuSO_4_, 1× Buffer additive provided with the kit, and either Alexa Fluor 488 dye azide or Cy5 azide) for 30 min at room temperature, protected from light and washed 3 times with 1% BSA in PBS. Then pellets were washed twice in 1x PBS, incubated with 2 μg/ml 4′,6-diamidino-2-phenylindole (DAPI) for 10 min, washed twice in PBS, mounted using 20 μl Prolong Diamond Antifade (Molecular Probes) and set overnight at room temperature.

Triple HA tagged *Pf*ORC proteins. Thin blood smears were fixed with 4% PFA for 15 min and quenched with 0.1 M Glycine for 10 min. After, they were permeabilised with 0.01% Triton X-100 and washed 3 times with PBS. Then samples were blocked in 1.5% BSA in PBS for 1 h at room temperature. Samples were incubated with the primary antibody monoclonal rat anti-HA (Roche, 3F10) diluted in 0.15% BSA at 1:2000, for 1 h, at room temperature, and then washed three times in PBS. Next, samples were incubated with the secondary antibody anti-rat Alexa 488 (Invitrogen) diluted in 0.15% BSA at 1:5000, for 1 h at room temperature and then washed three times in PBS. Before mounting using 20 μl Prolong Diamond Antifade (Molecular Probes), the DNA was stained with DAPI for 10 min and then washed twice in 1 PBS.

Slides were imaged using a Confocal ZEISS 880 FastAi microscope with a 63× immersion oil objective. Z-stacks were taken (0.16 μm inter-slice distance), initially processed with the default Airyscan Processing method and images in 2D were constructed from the maximum intensity projection on ImageJ. Colocalization analysis for each cell was done using the Jacop plugin from ImageJ.

### SDS-PAGE and western blot analysis

At least 500 μl of pelleted infected RBCs (∼4–5% schizonts) were collected by centrifugation at 1000 g and RBCs were lysed with 10V of 0.15% saponin. After a PBS wash, nuclei were isolated by sequential washes with different buffers. First, cells were lysed on ice with 1 ml of cell lysis buffer (CLB: 20 mM HEPES 7.9, 10 mM KCl, 1 mM EDTA, 1 mM EGTA, 0.64% NP40, 1 mM DTT and protease inhibitor cocktail) for 5 min. Next, the nuclear fractions (i.e. pellet) were washed three times in CLB and then lysed in 25 μl of Low Salt buffer (LSB: 20 mM HEPES 7.9, 0.1 M KCl, 1 mM EDTA, 1 mM EGTA, 0.64% NP40, 1 mM DTT and protease inhibitor cocktail) for 20 min at 4 °C. Finally, SDS-Laemmli buffer was added (final concentration 1×) to the nuclear lysates and sonicated to reduce viscosity. DTT was added to a final concentration of 100 mM and samples were boiled for 5 min before being loaded on a 10% SDS–PAGE gel. Gels were transferred onto PVDF membranes, blocked with 3% BSA in PBS for 1 h and then incubated overnight with the primary antibody monoclonal rat anti-HA (Roche, 3F10) diluted in 3% BSA at 1:2000. Following three washes with PBS-Tween 0.1%, the membranes were incubated with the secondary antibody anti-rat HRP (ab6845), washed three times with PBS-Tween 0.1% and developed with the Clarity Western ECL Blotting Substrate (BioRad).

### *Pf*ORC_1–2_ chromatin immunoprecipitation

Chromatin of *Pf*ORC1 and *Pf*ORC2 parasites was prepared as previously described (38) with minimal changes. Briefly, a total of 5 × 10^9^ infected RBCs (25 hpi ± 1 h) were cross-linked with methanol-free para-formaldehyde (final concentration 1%) at 37 °C for 10 min and then quenched with 0.125 M glycine for 5 min at 37 °C. Erythrocytes were washed once with PBS, lysed with 0.15% saponin and washed again with PBS. Parasites were then incubated on ice with in 4 ml of lysis buffer (10 mM HEPES pH 8.0, 10 mM KCl, 0.1 mM EDTA pH 8.0, and protease inhibitor cocktail) for 5 min, and then nuclei were disrupted with 150 strokes on a pre-chilled douncer homogenizer and pelleted by centrifugation for 10 min at 13 500 g, 4°C and resuspended in 3 ml of SDS lysis buffer (1% SDS, 10 mM EDTA pH 8.0, 50 mM Tris–HCl pH 8, and protease inhibitor cocktail). Next, the chromatin was sheared into fragments of 200–600 bp by sonication (Bioruptor; Diagenode) for 30 min (30 cycles, 15 s ON, 15 s OFF) and debris was removed by centrifugation for 10 min at 13 500 g, 4 °C. 10% of the sample was kept as ‘input’ and frozen until DNA purification.

For each immunoprecipitation, the chromatin was pre-cleared using 2 μg of rat IgG antibody for 2 h at 4 °C, with gentle rotation, followed by incubation with 20 μl of Magna ChIP Protein A/G Magnetic Beads (Sigma-Aldrich), for 2 h, at 4°C, with gentle rotation. The beads were harvested using a magnetic rack and further processed as the IP samples and later sequenced as ‘IgG control’. After, the pre-cleared chromatin was diluted ten-fold in ChIP dilution buffer (16.7 mM Tris–HCl pH 8.0, 0.01% SDS, 1.0% Triton X-100, 1.2 mM EDTA pH 8.0, 150 mM NaCl and protease inhibitor cocktail) and incubated overnight at 4°C, with 2 μg of monoclonal rat anti-HA antibody (Roche, 3F10), followed by a 2h incubation at 4°C with 20 μl of Magna ChIP Protein A/G Magnetic Beads (Sigma-Aldrich), with gentle rotation.

The IP and IgG fractions were washed at 4°C once with 1 ml of low salt wash buffer (20 mM Tris–HCl pH 8.0, 2 mM EDTA pH 8.0, 150 mM NaCl, 1% Triton X-100, 0.1% SDS), once with 1 ml high salt wash buffer (20 mM Tris–HCl pH 8.0, 2 mM EDTA pH 8.0, 500 mM NaCl, 1% Triton X-100, 0.1% SDS), once with 1 ml LiCl Wash Buffer (10 mM Tris pH 8.0, 1 mM EDTA, 250 mM LiCl, 0.5% NP-40, 0.5% sodium deoxycholate), and once at room temperature with 1 ml TE buffer (10 mM Tris pH 8.0, 1 mM EDTA pH 8.0). Immune complexes were eluted in 300 μl of elution buffer (10 mM Tris pH 8.0, 1 mM EDTA pH 8.0, 1% SDS) and incubated at 65°C for 30 min in an agitating thermal block (900 rpm 1 min, 30 s still).

Eluted co-immunoprecipitated IP DNA, IgG and input DNA were reverse-crosslinked by incubating at 65°C for 15 h. 300 μl of TE buffer were then added to each sample and RNAse A was added to a final concentration of 0.2 mg/ml, mixed by inverting and incubated for 2 h at 37°C. Proteinase K was finally added to a final concentration of 0.2 μg/ml and samples were incubated at 55°C for 2 h. DNA was precipitated using the phenol-chloroform method. Briefly, we added 600 μl of phenol:chloroform:isoamyl alcohol (P:C:IA—25:24:1) to the proteinase treated DNAs, and separated the aqueous phase through centrifugation at 10 000 g, for 5 min at 4°C. The aqueous layer was the transferred into a tube containing 600 μl of chloroform:isoamylalcohol (24:1), and spun at 10 000, for 5 min at 4°C. The aqueous layer was transferred to new tube containing and 800 μl of cold isopropanol. DNAs were pelleted by centrifugation at 20 000 g for 20 min at 4°C and washed twice with cold 80% EtOH, before being air-dried and resuspended in 10 μl of 10 mM Tris–HCl, pH 8.0. Each *Pf*ORC ChIP experiment was performed twice.

### *Pf*ORC_1–2_ library preparation, sequencing and analysis

DNA sequencing libraries were prepared using the MicroPlex Library Preparation Kit v3 (Diagenode) following the manufacturer's instructions and sequenced on a Hiseq2500 or NovaSeq SP flowcell, for a minimum of 5 million reads per sample.

Quality control of fastq files was performed using the FastQC software. Sequencing reads were aligned and mapped to the *P. falciparum* 3D7 genome (v.57 from PlasmoDB) using Burrows–Wheeler Alignment BWA MEM, to generate SAM files, keeping only uniquely mapped reads, which were then further processed using samtools to generate their corresponding BAM files. Peaks for the IP and IgG samples were called using MACS2 (*q*-value cutoff 0.05) with the input as control sample, with PCR duplicates removed. Bedtools suite was used to compute the intersection between any datasets throughout. The peak datasets for the two biological replicates of each ORC ChIP were intersected and only common peaks were kept. We next removed unspecific peaks from the resulting dataset by intersecting it with the IgG control. Non-specific regions were removed if *Pf*ORC peaks overlapped by >50 bp with IgG control peaks. A final filtering step to discard peaks smaller than 50 bp produced the final datasets that were used for further analysis and to generate the figures in this study.

For visual representation of the co-localisation between two datasets the deepTools suite was used. Matrix files were generated from bed and bigwig files using ComputeMatrix, and plotHeatmap was used for the visualisation of the *Pf*ORC1/2 overlap. The average enrichment profile of G4FS around ORC sites was determined and visualised using the plotProfile tool from bedtools after normalisation of peak scores. Fisher's exact test (bedtools) was used to test correlation between datasets (null hypothesis of independence). Association and overlap between the *Pf*ORC_1–2_ binding sites and post-translational modifications was investigated with the Jaccard similarity index (bedtools). The results of the pairwise comparisons with Jaccard were used to generate the heatmaps.

Inter origin distance (IOD) was computed with ‘bedtools closest’ within a single file with the ‘ignore overlaps (-io)’ and ‘ignore upstream (-iu)’ specifications to ensure that distances were computed between consecutive peaks. Distance between *Pf*ORC_1–2_ sites and the closest G4 site were computed with the standard mode of ‘bedtools closest’. Meta-gene plots were computed with the plotPeakProf2 (ChIPseeker), where each of the *P. falciparum* gene coordinates are scaled from 0 to 100% and converted into a segment of equal length. Each gene is flanked by a TSS (Transcription Start Site) and a TTS (Transcription Termination Site). Additionally, a region of equal dimensions to the gene size was added both upstream and downstream. Then, enrichment of *Pf*ORC_1–2_ was computed for the internal and flanking genes sequences and compared to a randomised control.

Empirical cumulative distribution functions (ECDF) were computed with the result of the bedtools closest calculations between the ChIP-seq datasets and the various query datasets and represented using the ECDF function from Hmisc on R. As a control, the genomic coordinates of each dataset were first randomly redistributed by shuffling them while keeping constant the number of sites per chromosome, and then they were subjected to the same analysis of computation of distances, as the experimental sites.

For associations of features with local gene expression levels (percentile) we used the timepoint t24 hpi from Chappell *et al.* (39).

### RNA-primed short nascent strand (SNS) DNA strand isolation, sequencing and analysis

SNS were purified as previously described (12,40) with minor modifications. Briefly, the cell number was increased to 10^8^ to ensure enough starting material. Synchronous parasites were harvested at 29 hpi (±1 h) and erythrocytes were lysed with 0.15% saponin and washed with 1× PBS. Next, the DNA was extracted in 20 ml of DNAzol. Then nascent stands were separated from genomic DNA on a 5 ml sucrose gradient. In each experiment three samples were prepared: (i) negative control—high molecular weight DNA devoid of SNS, (ii) SNS isolated in RNAse-free conditions and digested with λ-exo nuclease and, (iii) λ-exo background control: SNS isolated as in condition (ii) but subjected to a digestion with a cocktail of RNAses (RNase A + H, 50–100 μg/ml) to remove RNA primers at the 5′ end of the nascent strands prior to λ-exo nuclease digestion. This last sample was treated bioinformatically as an IgG control in a ChIP-seq experiment. All remaining procedures (volumes, reagents, incubation times) were performed as described in (12,40). The Illumina TruSeq ChIP Sample Prep Set A was used for preparation of sequencing libraries, which were sequenced either on a HiSeq 2000 or NovaSeq SP flowcell, for a minimum of 5 million reads per library.

Mapping, peak calling and the various correlation and statistics analyses were performed as described above for the ChIP-seq. Data from two sequenced experiments was merged.

### Nanoforkspeed (NFS) sample preparation and sequencing

For each culture with at least 5 × 10^8^ parasites at the different timepoints short pulses of 100 μM of 5-bromo-2′-deoxyuridine (BrdU) for 2 min were followed by a ‘chase’ of 1 mM of thymidine for 45 min. After, parasites were harvested and RBCs were lysed in 0.15% saponin/PBS and washed once in PBS. Then, the DNA was extracted with the Monarch^®^ HMW DNA Extraction Kit, following the manufacturer's instructions. A minimum of 3 μg of DNA was then purified using the Short Reads Eliminator kit (Circulomics) and libraries were prepared according to the ‘Native barcoding genomic DNA (with EXP-NBD104, EXP-NBD114 and SQK-LSK109)’ protocol provided by Oxford Nanopore. Each library was sequenced separately on the PromethION using R9.4.1 pore version. Basecalling, read mapping, fork directionality and speed analyses were performed as previously described (41) using the publicly available scripts deposited on https://github.com/LacroixLaurent/NanoForkSpeed.

The different datasets/coordinates used for the calculation of local fork speeds were as follows: centromere coordinates—ChIP-seq of the CenH3 protein (42); telomere coordinates—the first and last 3 kb of each chromosome; heterochromatin—regions marked by H3K9me3 (43) euchromatin—core genome without H3K9me3 tracks.

For the single molecule speed measurements, only the leading fork speed of the upstream origin was considered. Statistical analysis of the differences between fork speeds was performed with a Wilcoxon rank sum test using R.

## Results

### DNA replication timing in *P. falciparum*

In model eukaryotes, ORC recruitment takes place during the G1 phase of the cell cycle. In the absence of agents for chemically synchronising cells at a precise cell-cycle stage (44) and given the atypical nature of the S-phase in *Plasmodium* (Figure 1A), we used flow cytometry to first determine the timing of initial increase in DNA content in the population, and used it as proxy for average onset timing of the first S-phase, following RBC invasion. For that, a semi-synchronous population of *P. falciparum* 3D7 strain parasites was obtained by limiting RBC invasion to a 2 h window, after which new infections were prevented. Whilst before 26 h post invasion (hpi) the number of replicating parasites was very limited, at 26–28 hpi and 30–32 hpi, 9% and 43% of the parasite population, respectively, had doubled their DNA content (Figure 1B). Most parasites were, therefore, in a state akin to the late G1-phase at 25 hpi and, between 28 hpi and 30 hpi ∼34% of the population entered S-phase. To further confirm this timing, we generated a *P. falciparum* line carrying a viral thymidine kinase (TK+) that allows the parasite to incorporate nucleoside analogues such as 5-bromo-2-deoxyuridine (BrdU) and (5-ethynyl-2-deoxyuridine) EdU and used it to visualise replicating nuclei by immunofluorescence. As expected, at 25 hpi, EdU incorporation after 1 h treatment, was negligible, while at 29 hpi (i.e. the middle point of the time window 28–30 hpi) at least 18.9% of the population had actively replicated DNA in the preceding hour. At 35 hpi this proportion climbed to 96.6% (Figure 1C–E).

At 29 hpi most stained parasites had signal in the single nucleus (Figure 1C, E) while at 35 hpi, half-way through the asynchronous schizogonic S-phases (Figure 1A), each stained parasite had, on average, five EdU+ nuclei (Figure 1E). We thus defined 25 hpi ± 1 h as the window to study *Pf*ORC occupancy prior to DNA synthesis initiation, and 29 hpi ± 1 h as the window to study replication initiation events during the first S-phase.

### *Pf*ORC 1 and 2 binding sites are highly concordant and the complex often binds in clusters

**Figure 2.**
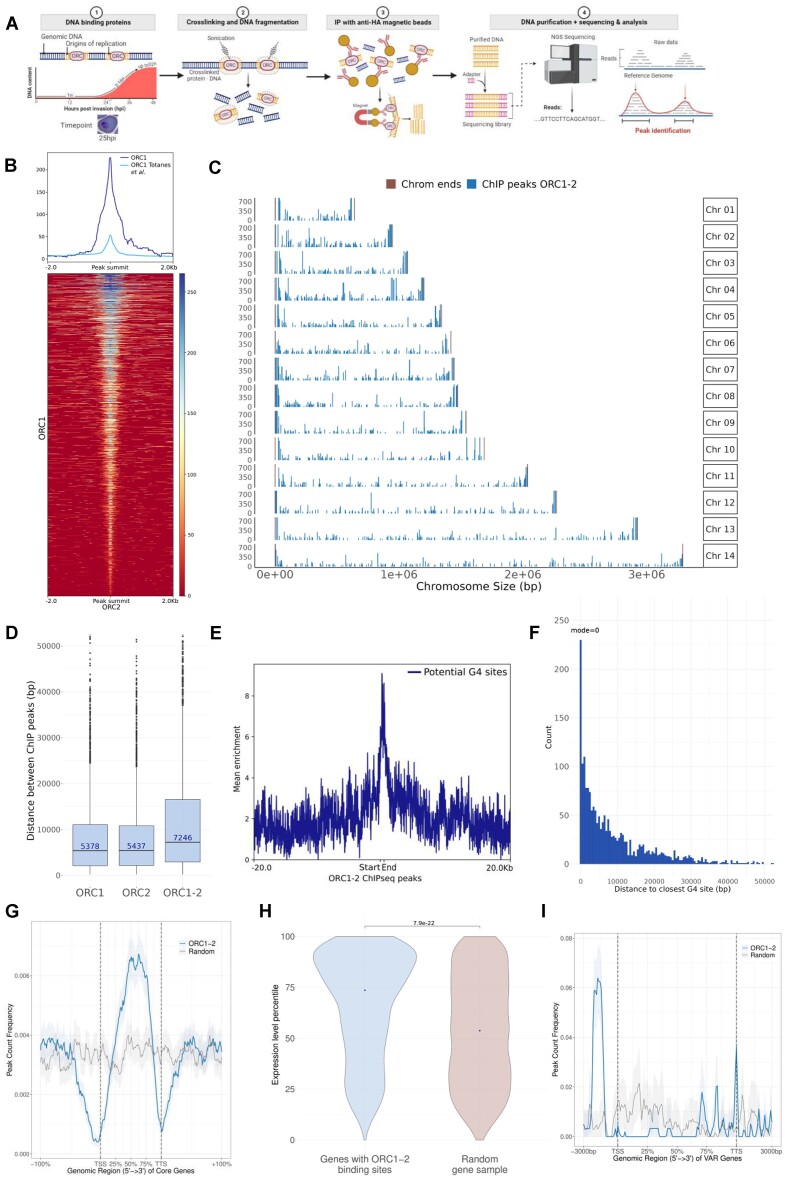
Profiling of *Pf*ORC1 and *Pf*ORC2 binding sites. (**A**) Schematic representation of the ChIP-seq protocol followed. Created with BioRender.com. (**B**) Enrichment of *Pf*ORC1 (this study's and Totanes *et al* 2023) around this study's *Pf*ORC2 binding sites. Heatmap displaying the enrichment of this study's *Pf*ORC1 peaks over this study's *Pf*ORC2 peaks flanked by 3 kb regions. The colour bar indicates the range of intensities based on ChIP enrichment, from red to blues for lower to high enrichment values. On the y axis, each row shows a *Pf*ORC2 ChIP peak centred in their summit and these are sorted in descending order based on the mean co-localisation of the datasets. (**C**) Genome wide distribution of the peaks common between *Pf*ORC1 and *Pf*ORC2. (**D**) Distance between *Pf*ORC binding sites (bp) measured for *Pf*ORC1 and *Pf*ORC2 and the *Pf*ORC_1–2_ intersected dataset. The median distance is shown. (**E**) Enrichment of G-quadruplex forming sequences (G4FS) around *Pf*ORC*_1–2_* sites. Peak lengths were scaled and are defined by ‘start’ and ‘end’ labels. (**F**) Histogram of the distances between a given *Pf*ORC*_1–2_* site and the closest G4FS; 13.6% of the total *Pf*ORC_1–2_ sites either overlapped, or were within 500 bp of a *Pf*ORC_1–2_ site. (**G**) Enrichment of *Pf*ORC*_1–2_* sites (blue) over scaled coordinates of *P. falciparum* genes. A randomized (control) dataset in shown in grey. Regions of equal length to each gene are included upstream and downstream the TSS and TTS, respectively. Both the TSS and TTS were depleted in *Pf*ORC sites. TSS: Odds ratio = 0.4 (anti-correlation), *P* value = 6.4 × 10^−54^; TTS: Odds ratio = 0.43 (anti-correlation), *P* value = 8.7 × 10^−25^). (**H**) Violin plots depicting the expression percentile of the genes displaying an enrichment of *Pf*ORC_1–2_ binding sites (n = 1347 genes) and a randomised dataset of equal number in brown. The result of a two-sided Wilcoxon test is shown. Expression percentiles were obtained from Chappell *et al.* (2020). (**I**) Enrichment of *Pf*ORC*_1–2_* sites (blue) over scaled coordinates of *var* genes.

To obtain a genome-wide map of ORC binding sites in the *P. falciparum* genome we performed ChIP-Seq of two different *Pf*ORC subunits—*Pf*ORC1 and *Pf*ORC2 (Figure 2A). Although phylogenetic analysis indicate that all six ORC subunits are likely present in the *Plasmodium* genome (45,46), we chose to focus on those two since the C-terminal region of both *Pf*ORC1 and *Pf*ORC2 were shown to be functional in *S. cerevisiae* complementation studies (47,48). Thus, we expected that, at least for these subunits, the replication initiator role would be conserved. We first generated triple hemagglutinin (HAx3) tagged lines for each of the subunits using CRISPR/Cas9 technology (Supplementary Figure S1a–c). We next confirmed that the insertion of the tag did not impact negatively parasite growth (Supplementary Figure S1d) and their correct expression by western blot (Supplementary Figure S1e). We also verified that at 25 hpi (late G1phase), both *Pf*ORC1 and *Pf*ORC2 were present in the nucleus (Supplementary Figure S1f, g).

Following ChIP-Seq, we identified 2693 peaks for *Pf*ORC1 and 2748 for *Pf*ORC2 at 25 hpi, in each case the intersection of two biological replicates (Supplemental Table S1). The binding profiles of *Pf*ORC1 and *Pf*ORC2 were highly concordant (*P* value < 1.6 × 10^−22^, Figure 2B, Supplementary Figure S2a, b). Indeed, most *Pf*ORC1 peaks overlapped with the *Pf*ORC2 dataset, which is consistent with the premise that these two proteins are in the same complex. In addition, although with lower scores and resolution, a *Pf*ORC1 ChIP-seq sample recently reported by Totañes *et al.* (49), showed a similar distribution to the *Pf*ORC datasets in this study Figure 2B). The overlapping set of *Pf*ORC_1–2_ binding sites were distributed throughout the genome (Figure 2C, Supplementary Figure S2c, d) at a median distance of 7246 bp from each other (Figure 2D), but as far apart as 106,655 bp, although only 10.9% of the sites (*Pf*ORC1: 3.6%; *Pf*ORC2: 3.3%) were separated by more than 30 kb. Since 25.6% of the *Pf*ORC_1–2_ sites were separated by less than 3 kb (*Pf*ORC1: 32.7%; *Pf*ORC2: 32.5%) we investigated if these complexes bind in clusters. Indeed, we detected 261 clusters with ≥ 3 binding sites within 10 kb (*Pf*ORC1: 557 clusters; *Pf*ORC2: 577 clusters), distributed throughout the genome that included a total of 635 *Pf*ORC_1–2_ sites (34.1%; Supplementary Figure S2e). In downstream analyses, we used only the peaks common to both *Pf*ORCs (Supplemental Table S1).

### Binding of ORC complex is not determined by sequence motifs but it is associated with putative G-quadruplexes

We could not find a consensus sequence in the *Pf*ORC_1–2_, akin to what was reported in Totañes *et al.* (49) for *P. falciparum* and in metazoans (10). Interestingly, we found that in *P. falciparum*, which bears one of the most skewed base pair compositions of any eukaryote (45), the *Pf*ORC_1–2_ peak sequences displayed an average GC content of 27% (Supplementary Figure S2f) —considerably higher than the genome wide content (19.3%); which was specifically seen around the peak summits (Supplementary Figure S2g). We reasoned that in *Plasmodium*, as in metazoans, ORCs might use other cues to select the binding sites such as GC-rich DNA secondary structures like G-quadruplexes. Indeed, the *Pf*ORC_1–2_ binding profile was strongly associated with the genome-wide distribution of G-quadruplex forming sequences (G4FS; G4H1.2 dataset from (50)) (Odds ratio = 3.04, *P* value = 2.1 × 10^−28^) and we found 342 G4FS that either overlapped, or were within 500 bp of a *Pf*ORC_1–2_ site (13.6% of the total *Pf*ORC_1–2_ sites; Figure 2E, F). Notably, high-score *Pf*ORC_1–2_ peaks either overlapped (top 1% *Pf*ORC_1–2_ peak score) or were in close proximity of a G4FS (Supplementary Figure S2h), suggesting that G-quadruplexes might play a role in the recruitment of the ORC complex in *P. falciparum* parasites.

### Strong transcription sites facilitate *Pf*ORC_1–2_ binding

To address the contribution of chromatin states to *Pf*ORC recruitment in a systematic and unbiased manner, we calculated the Jaccard index between the distribution of *Pf*ORC_1–2_ binding sites and available ChIP-seq datasets of histone PTM (captured at a similar timepoint) to measure their similarities. These comprised H3K9me3 (43), H3K9ac (51), H3K4me3 (51), H3K27ac (52), H3K18ac (52), H3K4me1 (52)) and the histone variant H2A.Z (52). The Jaccard index ranged from 0.005 to 0.063 (Supplementary Figure S2i) suggesting that PTMs on histone H3 are not likely to be directly involved in *Pf*ORC recruitment.

We then looked at the distribution of the *Pf*ORC_1–2_ sites relative to the coordinates of active genes at 25 hpi (39) and found that both the TSS and TTS (transcription termination sites) were depleted in *Pf*ORC sites. Instead, *Pf*ORC_1–2_ sites accumulated in the body of genes (Figure 2G; *P* value = 4.3 × 10^−45^) with a bias towards genes of higher expression (Figure 2H). Accordingly, the association between *Pf*ORC_1–2_ sites and the body of active genes was stronger when the expression percentile cut-off was increased to include only the 25% most expressed genes; these were three times more likely to contain a *Pf*ORC_1–2_ binding site that what would be expected by chance (Odds ratio = 2.9, *P* value = 1.82 × 10^−61^). In addition, we noted that telomeres, and in particular the gene bodies of transfer RNAs (tRNAs), which are unusually GC rich (median GC content: 34.5% and 55.4%, respectively), displayed a striking enrichment of *Pf*ORC_1–2_ sites (Supplementary Figure S2j).

### *Pf*ORC_1–2_ bind to the promoters of *var* genes

Antigenic variation allows parasites to evade the immune system and persist in the human host. A major player in this process is the *P. falciparum* variant surface antigen erythrocyte membrane protein 1 (*Pf*EMP1) which is encoded by the ∼60-member family of *var* genes (53). A system of mutually exclusive expression ensures that only a single *var* is expressed at a time while the others remain transcriptionally silent in H3K9me3 heterochromatin. *Pf*ORC1 was previously shown to bind telomeric and subtelomeric DNA including to the promoter of two *var* genes (54,55). We thus looked at the distribution of *Pf*ORC_1–2_ on *var* genes. The binding pattern was surprisingly different from that of the rest of the genome (Figure 2I). We detected a strong enrichment in *Pf*ORC_1–2_ binding sites on the TSS of *var* genes, specifically at around -1.5 kb. In addition, while there was also an enrichment on *var* gene bodies (44/61 genes), this was localised to three sharp and distinct regions including one at the very end of the coding sequence, spanning the TTS. Interestingly, this pattern was seen for both *Pf*ORC1 and *Pf*ORC2 and not just a feature of *Pf*ORC1 (Supplementary Figure S2k). In total 51 out 61 *var* genes contained *Pf*ORC binding sites. To ensure that this enrichment in the promoter was not a common feature of the heterochromatic environment, we looked at the *Pf*ORC_1–2_ distribution on the other H3K9me3 genes which include other virulence factors such as *rifin* and *stevor* genes. These displayed a *Pf*ORC_1–2_ depletion across the TSS and an enrichment in the gene bodies, akin to what was seen for core genes (Supplementary Figure S2l).

### Active origin mapping by SNS-seq reveals that ORC clustering is associated with origin firing

**Figure 3.**
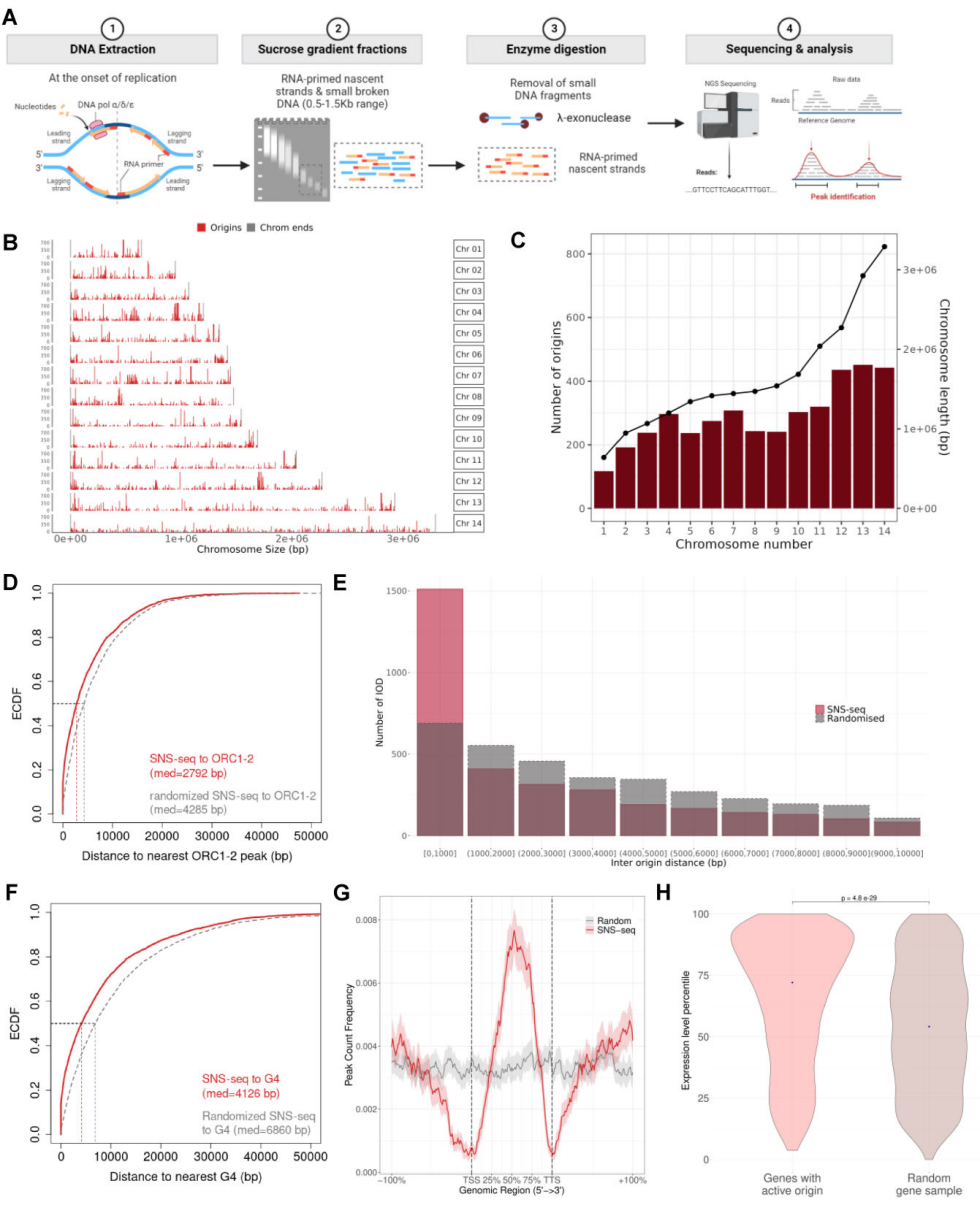
Genome-wide mapping of active origins of replication with SNS-seq. (**A**) Schematic of the experimental approach. Created with BioRender.com. (**B**) Genome wide distribution of SNS-seq origins. (**C**) Number of origins in each chromosome. Individual chromosome lengths are indicated by the black points. (**D**) Empirical Cumulative Distribution Function (ECDF) of the distances between SNS-seq origins and the closest *Pf*ORC_1–2_ site. Median distances are indicated. (**E**) Inter-origin distances between SNS-seq origins. Frequency of the various distances was calculated in 1 kb intervals. (**F**) ECDF of the distances between a given SNS-seq origins and the closest G4FS. (**G**) Enrichment of SNS-seq origins (red) over scaled coordinates of *P. falciparum* genes. A randomised (control) dataset in shown in grey. Regions of equal length to each gene are included upstream and downstream the TSS and TTS, respectively. Both the TSS and TTS were depleted in SNS-seq origins: TSS- Odds ratio = 0.191 (anti-correlation), *P* value = 3.48 × 10^−297^; TTS-odds ratio = 0.196 (anti-correlation), *P* value = 1.78 × 10^−283^. (**H**) Violin plots depicting the expression percentile of the genes displaying an enrichment of SNS-seq origins and a randomised dataset of equal number in brown. The result of a two-sided Wilcoxon test is shown. Expression percentiles were obtained from Chappell *et al.* (2020).

We next performed SNS-seq (Figure 3A) to map active ORIs and identified 4101 initiation sites (Supplementary Table S2), which were non-randomly distributed across individual chromosomes (Figure 3B). The number of origins mapped per chromosome roughly increased with chromosome length (Figure 3C). The inter-origin distances (IOD) were non-random and heterogeneous, with a median length of 2396 bp.

Binding of ORCs pinpoints where the replisome is to be recruited and, thus, where DNA replication begins. We therefore studied the relationship between the distribution of *Pf*ORC_1–2_ and the SNS-seq origins. There was a strong association between the two (odds ratio = 4.00, *P* value: 1.03 × 10^−123^) with an enrichment of nascent strand peaks around *Pf*ORC_1–2_ summits (Supplementary Figure S3a, b). Around 28% of the *Pf*ORC_1–2_ peaks overlapped with an SNS-seq peak (Figure 3D). However, since other systems reported that MCM cooperative binding and spreading after loading could lead to an expansion/shift of the initiation zone (56,57), we then re-calculated the overlap between the two datasets within a window of 2 kb and found that a striking 67% of the *Pf*ORC_1–2_ sites were within 2 kb of an origin (Supplementary Figure S3c).

A total of 1511 origins were separated by less than 1 kb (Figure 3E). We therefore wondered if there were clusters of origins, similar to what we had seen for *Pf*ORC_1–2_. We found 405 origin clusters with at least five origins within 10 kb, which were distributed throughout the genome (Supplementary Figure S3d). We found these SNS-seq clusters (hereon termed SNS clusters) to be strongly associated with the *Pf*ORC_1–2_ clusters (Odds ratio = 3.9; *P* value = 9.47 × 10^−21^). Indeed, nearly half of the *Pf*ORC_1–2_ clusters (43.7%, Supplementary Figure S3e) overlapped with the SNS clusters. In total, the *Pf*ORC_1–2_ clusters contained 778 SNS-seq origins (563 in the randomized cluster dataset), with an average of 3.4 origins per cluster (Supplementary Figure S3f). This strongly suggests that clustered licensing is more likely to lead to initiation events.

### Proximity to G4FS and strong transcription are associated with origin firing

The SNS-seq ORIs did not display any sequence specificity, but a similar increase in GC content relatively to the rest of the genome (32% vs 19.3%) was patent, being the highest around the summit of the SNS-seq peak (Supplementary Figure S3g, h). Accordingly, we detected a strong association with G4FS sequences (Odds ratio = 9.9; *P* value = 3.15 × 10^−294^, Figure 3F), which was stronger than what we had seen for the *Pf*ORC_1–2_ binding sites. A total of 978 origins (∼23.8%) had at least one G4FS within 500 bp (Supplementary Figure S3i).

Akin to what was seen for *Pf*ORC_1–2_, histone PTMs did not mark origins of replication (Jaccard index range: 0.002 to 0.126, Supplementary Figure S3j). On the other hand, origins were depleted in TSSs and TTSs, regardless of gene expression levels (Figure 3G), and the gene bodies of the 25% most expressed genes were strongly associated with initiation events (Odds ratio = 7.12; *P* value = 2.54 × 10^−178^; Figure 3H). Indeed, a large proportion of the genes of the top expression quartile (742 out of 1416) contained on average two origins. No enrichment was seen for genes of average or low expression levels. This indicates that while the association between origin licensing and gene bodies was significant irrespective of gene expression levels (albeit stronger with the top quartile), origin firing is likely enhanced by strong expression activity.

### Mapping of BrdU incorporation during DNA synthesis enables origin identification in *Plasmodium*

**Figure 4.**
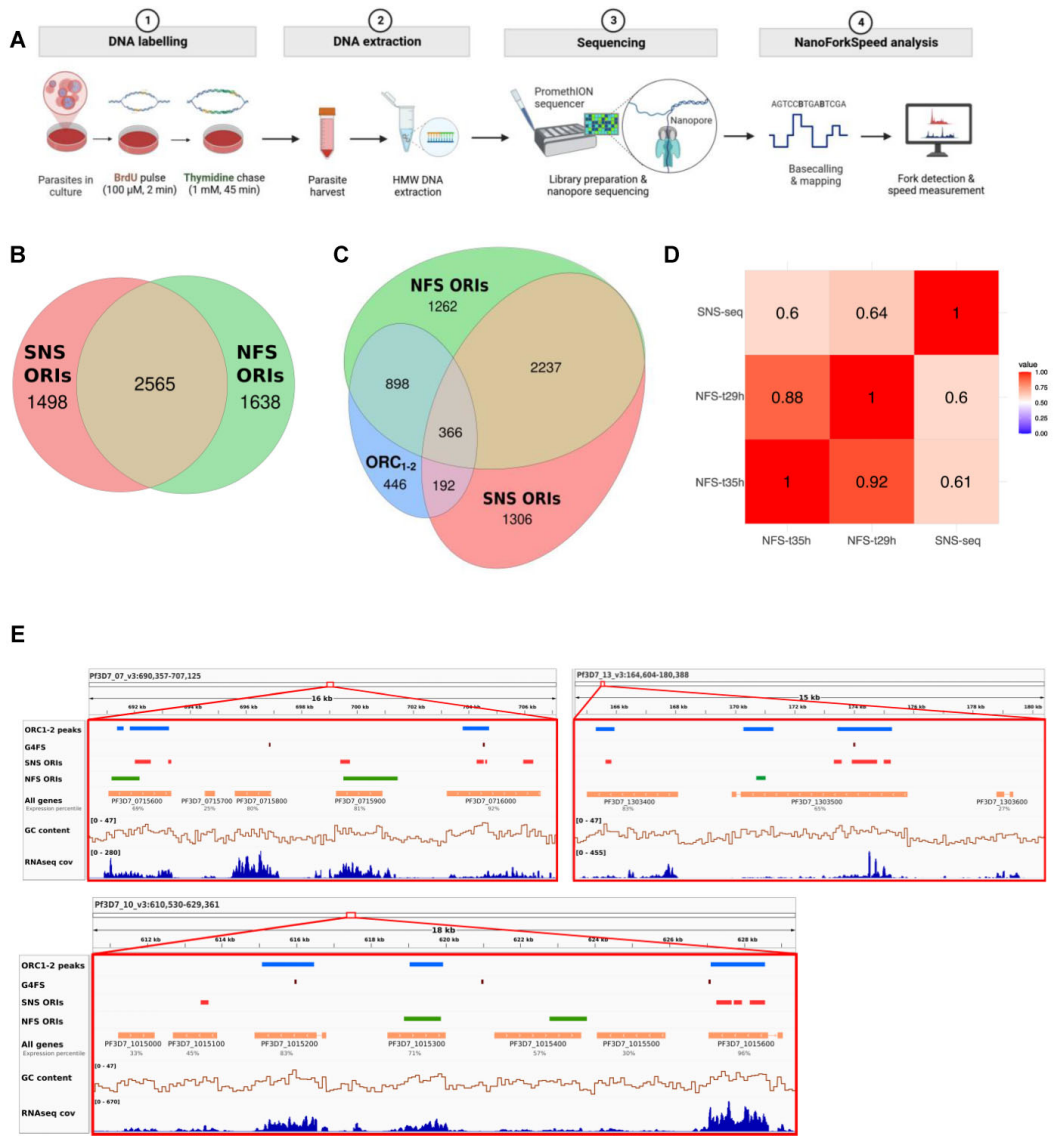
Mapping of active origins and fork speed with NFS. (**A**) Schematic of the experimental approach. Created with BioRender.com. (**B**) Overlap between the two active origins datasets (NFS and SNS-seq) (**C**) Overlap between the three datasets (active and licensed origins: NFS, SNS-seq *Pf*ORC_1–2_ binding sites). (**D**) Percentage of overlap between the active origin datasets. (**E**) IGV snapshots of the different datasets depicting a section of the genome on chromosomes 7, 10 and 13. From top to bottom: *Pf*ORC_1–2_ ChIP-seq peaks, G-quadruplex forming sequences (G4FS) coordinates from Gazanion *et al.* (2020), SNS-seq origins (red), NFS origins at 29 hpi (green), encoded genes and their expression percentile (Chappell *et al.* 2020). The last two tracks show the corresponding genomic GC content (bin size = 100 bp), as well as local RNA-seq coverage.

Using fluorescent reporters and microscopy, Klaus *et al.* reported that the first S-phase is significantly longer than the ensuing ones (29). We reasoned that this could be due to (i) a lower number of origins or (ii) a slower fork speed in the first S-phase. To address this question, we employed the NanoForkSpeed (NFS) method to map and orientate replication forks, which permits not only mapping of initiation events and replication forks directionality profiles, but also measurements of fork speed (41). For that we subjected the thymidine kinase-expressing (TK+) *P. falciparum* line to short pulses (2 min) of 100 μM BrdU, followed by a 10-fold thymidine chase (45 min) at two different stages of schizogony: 29 hpi (onset of S-phase) and 35 hpi (mid schizogony). The labelled DNA was then sequenced with Oxford nanopore technologies (ONT; Figure 4A). We obtained the expected BrdU signals (41) with abrupt upward slopes downstream of segments of zero BrdU content (i.e. initiation of the BrdU pulse), and then longer downward slopes of progressively lower BrdU intensity (*i.e*. incorporation of BrdU during the chase, which is progressively diluted by the thymidine chase; (Supplementary Figure S4a)). We also confirmed that any modified DNA bases present in the genome did not interfere with the BrdU calling (Supplementary Figure S4b). Directionality of the forks was subsequently inferred (Supplementary Figure S4a) and this information was used to map initiation and termination events. An initiation event was defined as the region between two divergent forks (in the same read) and a termination event as the region between two converging forks (in the same read).

For the timepoints 29 hpi and 35 hpi, we mapped ∼7.6 M and 13.6 M reads, of which 47,421 and 43,801 contained at least one fork and, 3722 and 3232 contained at least one initiation event, respectively. The median size of a read containing at least an initiation event was 37,816 bp (29 hpi) and 37,548 bp (35 hpi; Supplementary Figure S4c). A total of 54,590 and 50,117 forks, which corresponded to a similar density between timepoints of 2.34 and 2.15 forks per kilobase of the genome, allowed us to map 3786 (Supplementary Figure S4d) and 3286 (Supplementary Figure S4e) active origins of replication and 3360 and 3015 termination events, for 29 hpi and 35 hpi, respectively (Supplementary Table S3).

Given that different origin mapping methods often show limited overlap (30,58–60) due to technology biases, we compared the distribution of the 29 hpi NFS initiation events with the *Pf*ORC_1–2_ binding sites and SNS-seq origins. Notably, about two thirds of the *Pf*ORC_1–2_ and SNS-seq sites overlapped with the initiation regions of t29 hpi NFS (Figure 4B–E), demonstrating the complementarity of the methods and robustness of the datasets. On the other hand, 90% of the *Pf*ORC_1-2_ clusters contained between one and 18 NFS origins (mean = 3.88) and comparison of the t29 hpi NFS IODs for each chromosome showed an overall agreement with the SNS-Seq IODs (Supplementary Figure S5a). If we restricted the width of the initiation regions to ±1.5 kb around the middle point between converging forks, the different datasets remained highly concordant (*P* value(*Pf*ORC_1–2_)= 1 × 10^−2^; *P* value (SNS-Seq) = 2.6 × 10^−3^; Figure 4D). Accordingly, a similar depletion in TSSs and an enrichment in gene bodies, with a bias towards genes of higher expression, was seen for the NFS origins (Supplementary Figure S5b, c).

We next searched for clustered t29 hpi NFS origins (±1.5 kb around the middle point) and found 489 clusters of at least 5 origins within 10 kb (Supplementary Figure S5d), a very similar number to what was found for the SNS-Seq origins. The distribution of the t29 hpi NFS clusters correlated with the *Pf*ORC_1–2_ (*P* value = 3.1 × 10^−4^) and SNS-Seq (*P* value = 1.61 × 10^−5^) clusters with about one third overlap, in both cases (Supplementary Figure S5e, f).

Although a link with G4FS was not evident in the NFS dataset, we concluded that overall, these different mapping methods are valid and concordant for the study of replication origins in *Plasmodium* (Figure 4E). In addition, as comparable fork density and number of initiation events were detected between timepoints, we considered that the difference in duration of the S-phases reported by Klaus *et al.* is unlikely to be due to a difference in number of active origins.

### Different chromatin environments impact local fork speed

**Figure 5.**
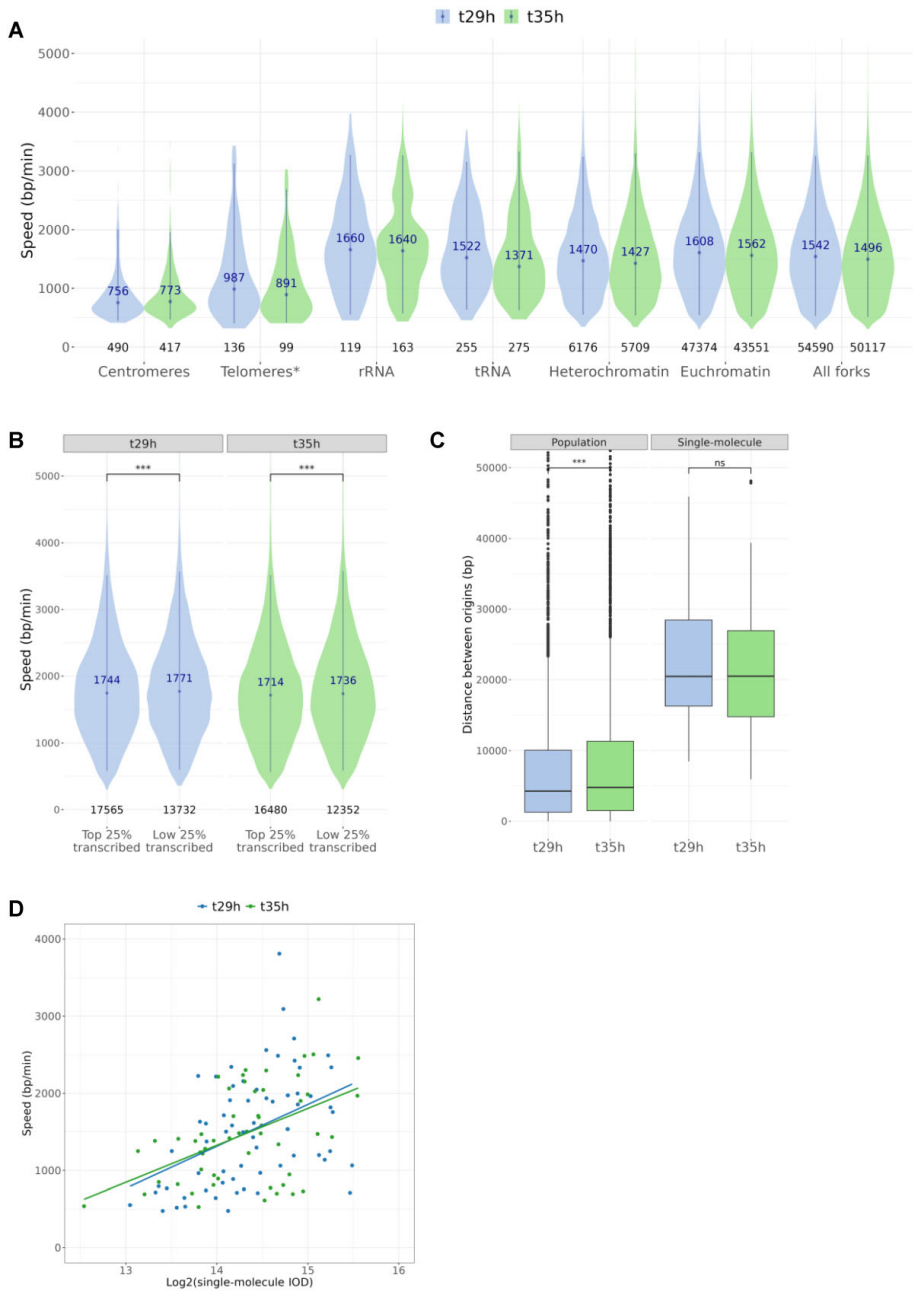
Mapping of active origins and fork speed with NFS. (**A**) Local fork speeds detected at t29h and t35h, over specific genome features or gene families. *Telomeres were defined as the first and last 3 kb of each chromosome as per suggested by Figueiredo *et al.* (**B**) Variation of fork speed when travelling over highly or lowly expressed genes. Median velocities are shown. Wilcoxon test *P*-value (t29h) = 0.00016 and *P*-value (t35h) = 0.00093. (**C**) Inter-origin distance calculated for the total population data (*P*-value = 4.7 × 10^−4^) or from sequencing reads containing multiple initiation events which could have only come from one nucleus (*P* value = 0.64). (**D**) Relationship between single cell IOD and the respective leading strand fork speed. These two variables were found to be strongly associated (Two-way ANOVA, *P* value = 9.2 × 10^−6^), and the factor timepoint was found not to significantly influence them (two-way ANOVA, *P* value = 0.7).

Fork speed can be calculated as the length of the labelled section divided by the labelling time (*i.e*. 2 min). Accordingly, the NFS algorithm captures the length of the section between the starting and ending points of the steep slopes (Supplementary Figure S4a), in other words the track produced during the BrdU pulse (41). The median genome wide speed was 1542 bp/min for 29 hpi and 1496 bp/min for 35 hpi (Figure 5a), in agreement with the DNA combing data reported by Stanojcic *et al.* of 1.4 kb/min for semi-synchronous early replicating parasites, and 1.2 kb/min for semi-synchronous late replicating parasites (61). Lagging strand forks were slightly slower than leading strand forks at 35 hpi (1486 bp/min and 1510 bp/min, respectively; *P* value = 4.9 × 10^−6^) but of seemingly similar speeds at 29 hpi (Supplementary Figure S5g).

We next explored local fork speed variations by looking at specific regions of the genome: centromeres, telomeres, rRNAs, tRNAs, heterochromatin (H3K9me3 marked) and euchromatin (H3K9me3 negative; Figure 5A). Fork speed was drastically reduced in centromeres in both timepoints with a median speed of 756 bp/min and 773 bp/min, respectively. This decrease in speed was not due to a decrease in read coverage (Supplementary Figure S5h). Similar speeds were measured for forks travelling over the telomeres (987 bp/min and 891 bp/min). The speed was the highest in rRNAs (1660 bp/min and 1640 bp/min) while tRNAs displayed a median speed slightly below the genome average (1522 bp/min and 1371 bp/min). We also noted a difference in speed when comparing H3K9me3 rich (heterochromatin) with H3K9me3 depleted (euchromatin) regions, with the former displaying significantly slower fork speeds in both timepoints (*P* value_(29 hpi)_ = 5.7 × 10^−36^, *P* value_(35 hpi)_ = 1.4 × 10^−29^). Finally, gene expression level was also a factor that appeared to slightly influence fork speed, with lower expression being associated with higher speeds both at 29 hpi and 35 hpi (Figure 5B).

### Single molecule measurements reveal a link between fork speed and inter-origin distance

Calculating IODs from bulk methods like the SNS-seq is complicated by the fact that they reflect population averages. In the NFS method, however, although the input is a mixed population of cells, it relies on single molecule information, each originating from a single cell. As a result, by using reads containing multiple initiation events we were able to calculate IODs between origins that could have only come from individual nuclei. We found 64 and 52 reads with two or three origins in the 29 hpi and 35 hpi datasets, respectively. While reads containing one initiation event were on average 37 kb, the ones containing ≥ 2 initiation events were on average 56 kb and 57 kb, for 29 hpi and 35 hpi, respectively. The single cell IODs were considerably longer than the IODs previously calculated for the total population and they were similar between timepoints—on average 22,823 bp at 29 hpi and 21,943 bp at 35 hpi (Figure 5C). Interestingly, when we looked at the speed of the leading forks and the distance that they needed to travel we found a significant link between these single-molecule-IODs and the respective leading fork speed, with longer IODs being associated with faster forks, independently of the timepoint analysed (*P* value = 9.2 × 10^−6^, Figure 5D).

By combining single-molecule speed and IOD information we concluded that, in the absence of fork stalling, asexual *Plasmodium falciparum* parasites could complete one round of DNA replication in less than 15 min, both at the beginning or middle of schizogony. It is thus unlikely that the reason for the faster replication cycles after the first S-phase is linked to origin density or fork speed.

## Discussion

Much of the diversity in the domain Eukarya lies in microbes, many of which display stark differences in core biology from model, predominantly opisthokont, organisms. Though most of our understanding of DNA replication in eukaryotes has focused on yeast and metazoans, a few recent studies have started to explore nuclear replication in *Plasmodium falciparum* parasites (27,29,49,62). By measuring DNA content increase through flow cytometry and by timing the incorporation of thymidine analogues we determined that about one third of a semi-synchronous culture of parasites initiates the first DNA synthesis phase at around 29 hpi, which matches onset timing estimates reported in the 80s (34).

ORIs of unicellular organisms such as yeast are typically located in regions of the genome that are relatively AT-rich. However, in the case of the AT-rich genome of *P. falciparum*, *Pf*ORC proteins tend to bind to regions with a relatively high GC content. Surprisingly, given the difference in GC content of these genomes (19% in *P. falciparum*, 38% in *S. cerevisiae*), what was perceived as AT-rich (*i.e*. low GC) in yeast, actually corresponded to GC-rich in the *P. falciparum* genome, suggesting an evolutionarily conserved requirement for a specific GC content in ORC binding. In some cases, *Pf*ORC_1–2_ binding was associated with G4FS. Whether G-quadruplexes have a role in the recruitment of ORC proteins or simply provide nucleosome excluded regions that in turn allow *Pf*ORC binding remains to be clarified, but a strong link between G-quadruplexes and ORIs has been demonstrated in mammalian cells (12). In contrast with the metazoan model, where epigenetic features such as PTM of histones like H3 acetylation and H3K4 methylation influence ORC recruitment (15,63,64), analysis of the *P. falciparum* PTM datasets available suggested that PTMs are unlikely to be directly involved in the recruitment of *Pf*ORC_1–2_. However, the fact that these marks are rather dynamic combined with a potential difference in parasite age of the different datasets might have led to missed biological insights in this analysis. Transcription on the other hand, was found to be a strong determinant of ORIs in *P. falciparum* but not in the same way as in human cells. Active TSS are necessary, and sufficient for ORC binding in humans (63,65), perhaps because the mechanisms that specify TSSs also determine the location of the origins of replication. However, in *P. falciparum* we observed a depletion of ORIs on both TSS and TTS. Instead, they were enriched on transcriptionally-active gene bodies. This was surprising as it could be a source of replication/transcription conflicts, but it might be a consequence of the extreme AT-richness of the intergenic regions/promoters (90–95%). In this scenario, while a similar recruitment mechanism could exist in *Plasmodium*, a recruitment into the more GC balanced regions of the gene bodies would provide a more stable platform for the assembly of the replisome.

Other than a role in DNA replication, ORCs have been shown to be involved in transcriptional silencing. For instance, in *S. cerevisiae* silencing of the *HM* mating type loci relies on the binding of ORC (66,67), and in *Drosophila*, *Xenopus* and mammals, both ORC1 and ORC2 were shown to interact directly with heterochromatin protein HP1 (55). Similarly, *Pf*ORC1 was previously implicated in gene silencing due to an association with the histone deacetylase *Pf*Sir2 (54). The *var* gene family, which is under strict regulatory mechanisms that ensure mono-allelic expression, displayed a dramatically different pattern of *Pf*ORC_1–2_ binding. Unlike any other studied region of the genome, *var* genes, which are present in heterochromatic regions, exhibited an enrichment in *Pf*ORC_1–2_ sites on the TSS. This binding pattern might thus reflect a conserved role of *Pf*ORCs in gene silencing or regulation of mono-allelic expression in *P. falciparum* parasites.

Mapping of active origins by sequencing short nascent strands yielded a total of 4101 active sites in the parasite population at ∼29 hpi — a similar number to what was described in the parasite *Leishmania major* (∼5100 sites) using the same technique and whose genome is of comparable size (68). Our dataset revealed that most *Pf*ORC_1–2_ binding sites were located within 2 kb of an active site of initiation. The genome wide distribution of short nascent strands was similar to the one of *Pf*ORC_1–2_ binding sites, with a stronger association with G4FS, and a similar depletion of TSS and enrichment on gene bodies. Strikingly, we found that in *P. falciparum* strong transcription might enhance origin firing as active sites were mostly, and strongly, associated with the top expression quartile genes. Activation of origins in these parasites might thus require spatial-temporal coordination of the different polymerase complexes as well as careful maintenance of the number of ORIs to activate — as few origins as possible to avoid conflicts with transcription, but enough to allow replication of the entire genome in the absence of genome instability. Additionally, we saw that around 66% of ORIs were grouped in 405, 10 kb-long clusters or initiation zones. The existence of these clusters may reflect general preferential initiation regions in the genome. Each origin in the cluster may not be active in the same cell but may represent a population propensity to initiate in these regions.

In metazoans, the number of ORC sites vastly exceeds the number of initiation sites in a given cell cycle (10). This seemingly excessive licensing has a critical role in the maintenance of genome stability as the extra (dormant) licensed sites can be activated and become additional replication start sites, to protect the cells from replicative stress and/or rescuing stalled forks. In this study this was not the case, perhaps due to technical limitations or because we used very stringent analysis parameters to ensure a high confidence dataset of *Pf*ORC binding sites — only common sites to four datasets were used in the analyses (intersection of ChIP-seq sites of two different proteins, each with two different replicates). Nevertheless, a recent study by Totañes *et al.* reported ∼9000 *Pf*ORC1 binding sites (49), almost five times more than this study's *Pf*ORC_1–2_ dataset, which were distributed about every kilobase. Notably, perhaps due to a high noise/signal ratio, the authors did not find a correlation with active origins and presented a negative correlation with transcription. Regardless, based on the available data, it seems unlikely that *P. falciparum* parasites display a ratio licensing/activation comparable to the metazoan system. We cannot, however, exclude the existence ORC independent replication initiation with perhaps the participation of other initiator proteins, given that more than half of the genome of malaria parasites lacks functional annotation and that a comprehensive study of the pre/replicative complex of *P. falciparum* parasites is yet to be performed.

As Oxford nanopore sequencing technology allows base analogues to be recognised from the specific electric profile signatures that they generate when the DNA strand translocates through the pore, this is an excellent tool to study active DNA replication (41,69) especially in organisms with relatively small genomes like *P. falciparum* (∼24 Mb). Indeed, the overall agreement between the NFS data and *Pf*ORC_1–2_ and SNS-seq sites showcases their validity for ORI mapping in *Plasmodium* parasites. These data also showed that the genome wide median speed of replication forks was of around 1.5 kb/min, with a difference of 46 bp/min (3%) between the two timepoints. Whether this difference is biologically meaningful remains unclear. While this speed was on par with previous DNA combing estimates (61) it contrasted to the speed measurements in the Totañes *et al.* study of 0.589 kb/min. A possible explanation for this discrepancy is the use of a very long pulse (15 min (BrdU + EdU)) in the latter study. Longer pulses increase the probability of forks to merge thus decreasing their detection rate, particularly in the case of faster forks. As a result, with a longer pulse, faster forks are likely missed and thus the median or mean speed is slower.

The widespread use of techniques like DNA combing which allow single cell measurements instead of population averages, has revealed that IODs tend to vary with the species, being very large in trypanosomatids parasites such as *Trypanosoma brucei* (160 kb) and *Leishmania mexicana* (226 kb) (70,71) or shorter in other systems, for instance 46 kb in yeast (72), 137 kb in mouse cells and 73 kb in Drosophila (10). Analysis of NFS reads with multiple origins allowed the estimation of single nuclei IOD, which ranged from 9 to 46 kb. Although comparable, these were a slightly shorter than what was previously reported on a DNA combing study, which for 24–30 hpi parasites was on average 68.5 kb (61). It is, however, possible that this discrepancy arises from the limited range of nanopore read length which was in this case of ∼99 kb.

Using our data, we also tried to understand the reason why the first S-phase would be considerably longer than the subsequent ones. Indeed, using PCNA1::GFP as a *nuclear cycle sensor*, Klaus and colleagues determined that the first S-phase takes ∼50 min and the ensuing rounds ∼35 min (29). Our data indicates that during schizogony, in the absence of roadblocks, replication of the genome is possible in ∼15 min. Given that there are similar densities of active origins, IODs and fork speeds between the two analysed timepoints, we speculate that there might be a higher rate of fork stalling in the first S-phase compared to the subsequent rounds.

## Conclusions

In this study, we provide a comprehensive study of the origins of replication in the human malaria parasite *P. falciparum*. We found that the ORIs of *P. falciparum* display some features common to the mammalian system such as lack of sequence specificity, GC-richness and association with G4FS. However, we also discovered some unusual features in *Plasmodium* such as an association of ORIs with gene bodies of strongly transcribed genes, and a depletion from TSS/TTS. Such unorthodox origin usage provides insight into the evolution of origin specification and activation amongst eukaryotes. In this case, it is perhaps a consequence of the extreme AT-richness of the genome, tailored by evolution and the parasitic lifestyle (39,73,74). Overall, this represents an important step towards understanding the replicative process that allows these parasites to expand their population by several orders of magnitude in a matter of days.

## Supplementary Material

gkad1103_supplemental_files

## Data Availability

All sequencing data underlying this article are available in the ENA database under accession code PRJEB62206. The analysis pipeline underlying this article is available in Zenodo at https://doi.org/10.5281/zenodo.10039720.

## Notes

Present address: Paulina Prorok, Department of Biology, Technical University of Darmstadt, 64287 Darmstadt, Germany.
