# Supplementary material for: The genetic landscape of origins of replication in P. falciparum
Source: Nucleic Acids Res. 2023 Dec 1;52(2):660–76. doi: 10.1093/nar/gkad1103 (PMC10810204; doi:10.1093/nar/gkad1103)
Supplement: gkad1103_supplemental_files [file gkad1103_supplemental_files.zip › sup_Figs_NAR_Resub.pdf]

S1

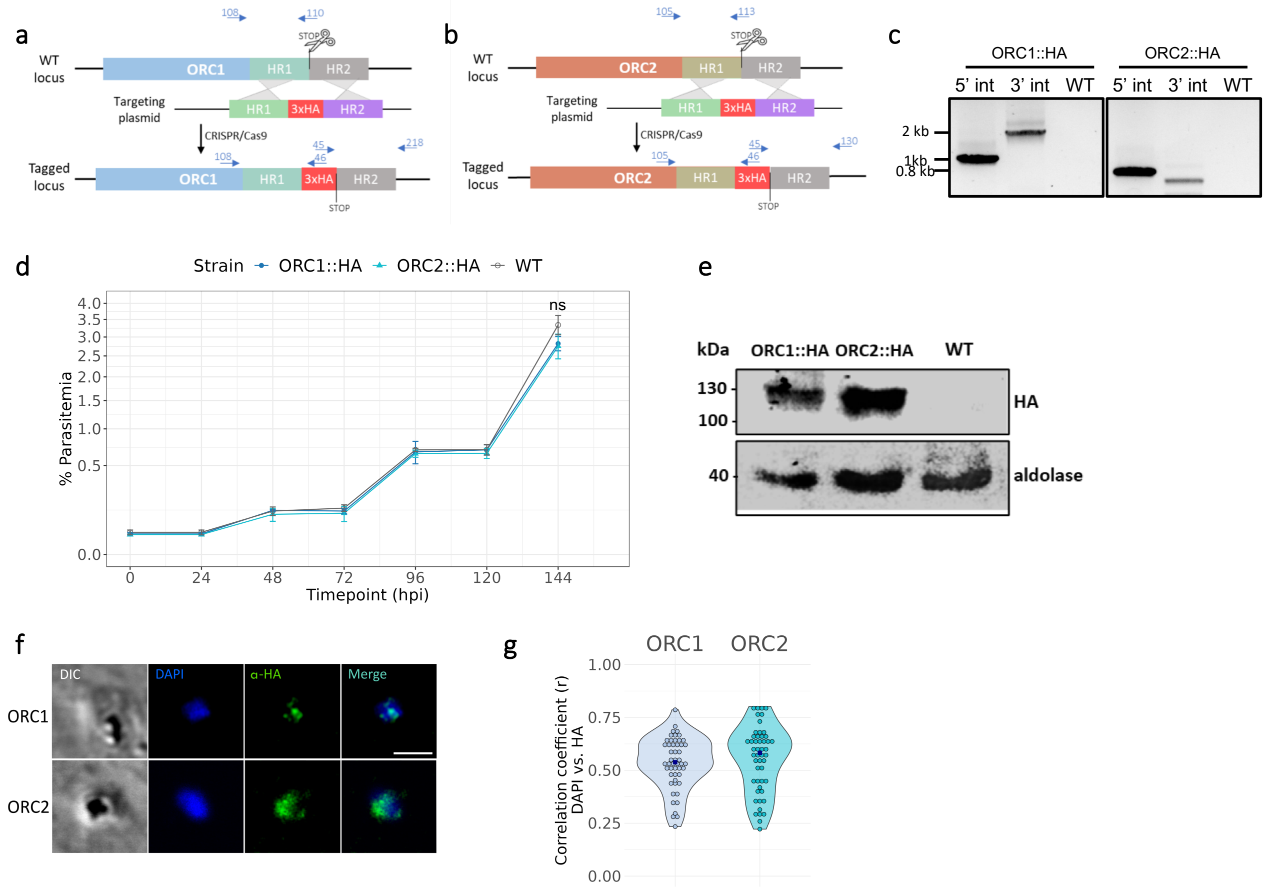

Supplemental figure 1. Generation of 3xHA tagged *Pf*ORC1 and *Pf*ORC2. a, b) Schematics illustrating the plasmids construction for the endogenous C-terminus tag of *Pf*ORC1 **(a)** and *Pf*ORC2 **(b). c)** Confirmation of the presence of the 3xHA tag by PCR. Primers and fragment sizes are, from left to right, 108/46: 1076 bp, 45/218: 1815 bp, 108/110: 1024 bp for ORC1; and 105/46: 798 bp, 45/130: 669 bp, 105/113: 750 bp for ORC2. The reactions "5' int" and "3' int" refer to integration PCRs targeting the 5' or 3' sites, respectively, while "WT" refers to the wild type locus. **d)** Growth curves of both *Pf*ORC mutants alongside a WT (*Pf*3D7) control, showing comparable growth between the parasite lines. ns: not significant. **e)** Western blot confirming correct expression and size of *Pf*ORC1::HA and *Pf*ORC2::HA. The predicted sizes were 124 KDa for *Pf*ORC1::HA and 101 KDa for *Pf*ORC2::HA. α-aldolase was used as a loading control (40 KDa). **f)** Localisation of *Pf*ORC1::HA and *Pf*ORC2::HA. DNA is labelled in blue with DAPI, *Pf*ORC::HA signal is shown in green. Scale bar represents 2 µm. **g)** Quantification of the Pearson's correlation coefficient between the fluorescent signal of the HA tag and the DAPI. At least 50 parasites were measured in Fiji with the Jacop plugin. Median r values are indicated with a blue mark (r=0.54 for *Pf*ORC1 and r=0.58 for *Pf*ORC2).

S2

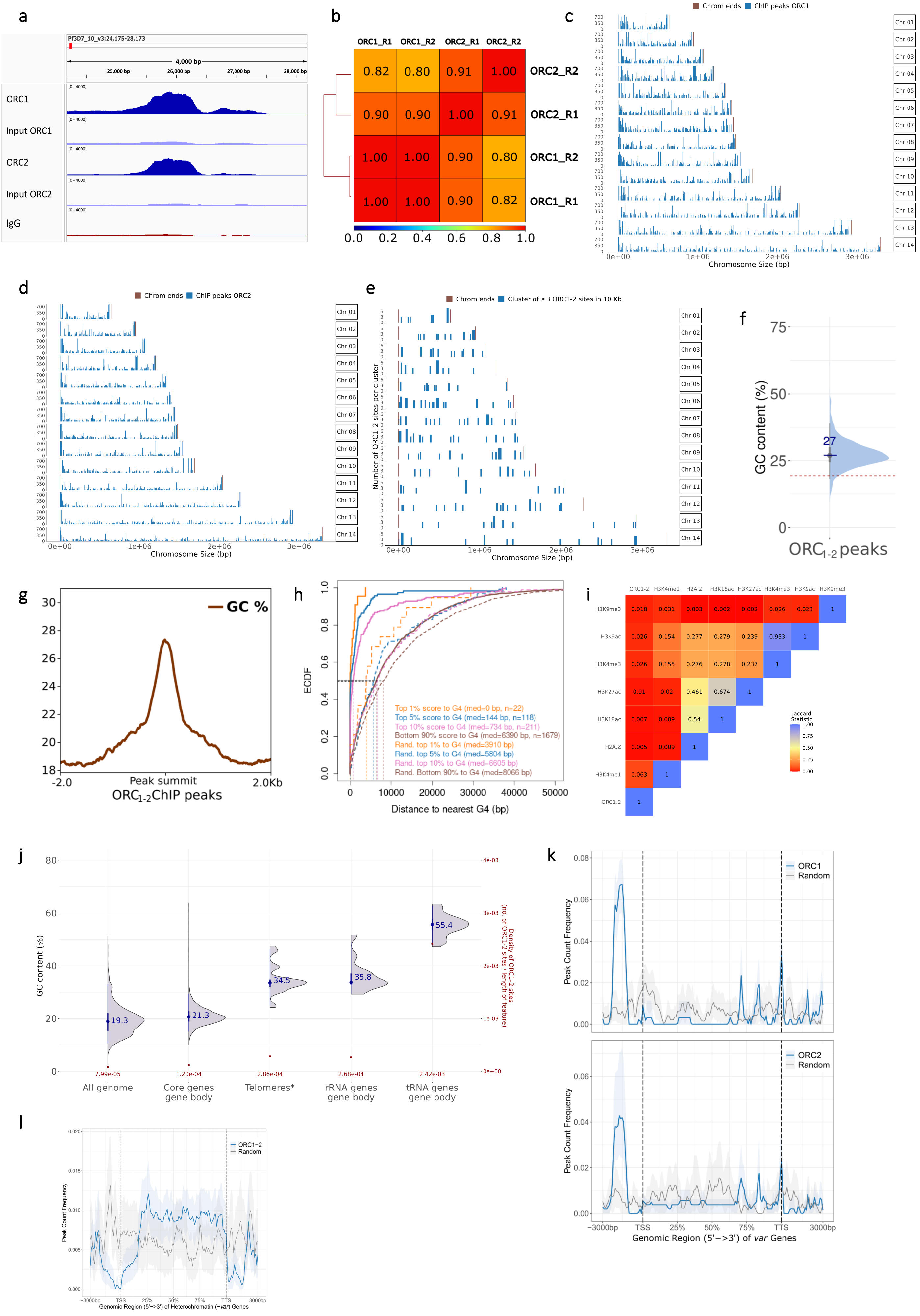
a
Pf3D7_10_v3:24,175-28,173
4,000 bp
25,000 bp
26,000 bp
27,000 bp
28,000 bp
[0 - 4000]
ORC1
Input ORC1
ORC2
Input ORC2
IgG
b
ORC1_R1
ORC1_R2
ORC2_R1
ORC2_R2
0.82 0.80 0.91 1.00
0.90 0.90 1.00 0.91
1.00 1.00 0.90 0.80
1.00 1.00 0.90 0.82
0.0 0.2 0.4 0.6 0.8 1.0
c
Chrom ends
ChIP peaks ORC1
Chr 01
Chr 02
Chr 03
Chr 04
Chr 05
Chr 06
Chr 07
Chr 08
Chr 09
Chr 10
Chr 11
Chr 12
Chr 13
Chr 14
0e+00
1e+06
2e+06
3e+06
Chromosome Size (bp)
d
Chrom ends
ChIP peaks ORC2
Chromosome Size (bp)
e
Chrom ends
Cluster of ≥3 ORC1-2 sites in 10 Kb
Number of ORC1-2 sites per cluster
Chromosome Size (bp)
f
GC content (%)
27
ORC1-2 peaks
g
GC %
Peak summit
-2.0
2.0Kb
ORC1-2ChIP peaks
h
ECDF
Top 1% score to G4 (med=0 bp, n=22)
Top 5% score to G4 (med=144 bp, n=118)
Top 10% score to G4 (med=734 bp, n=211)
Bottom 90% score to G4 (med=6390 bp, n=1679)
Rand. top 1% to G4 (med=3910 bp)
Rand. top 5% to G4 (med=5804 bp)
Rand. top 10% to G4 (med=6605 bp)
Rand. Bottom 90% to G4 (med=8066 bp)
Distance to nearest G4 (bp)
i
ORC1-2
H3K4me1
H2A.Z
H3K18ac
H3K27ac
H3K4me3
H3K9ac
H3K9me3
H3K9me3: 0.018 0.031 0.003 0.002 0.002 0.026 0.023 1
H3K9ac: 0.026 0.154 0.277 0.279 0.239 0.933 1
H3K4me3: 0.026 0.155 0.276 0.278 0.237 1
H3K27ac: 0.01 0.02 0.461 0.674 1
H3K18ac: 0.007 0.009 0.54 1
H2A.Z: 0.005 0.009 1
H3K4me1: 0.063 1
ORC1.2: 1
Jaccard Statistic
1.00
0.75
0.50
0.25
0.00
j
GC content (%)
Density of ORC1-2 sites (no. of ORC1-2 sites / length of feature)
19.3
21.3
34.5
35.8
55.4
7.99e-05
1.20e-04
2.86e-04
2.68e-04
2.42e-03
All genome
Core genes gene body
Telomeres*
rRNA genes gene body
tRNA genes gene body
k
ORC1
Random
ORC2
Random
Peak Count Frequency
-3000bp
TSS
25%
50%
75%
TTS
3000bp
Genomic Region (5'–>3') of var Genes
l
ORC1-2
Random
Peak Count Frequency
Genomic Region (5'–>3') of Heterochromatin (–var) Genes

Supplemental Figure 2. Distribution of *Pf*ORC1 and *Pf*ORC2 binding sites. **a)** IGV snapshot showing an example of overlapped *Pf*ORC1 and *Pf*ORC2 peaks. **b)** Heatmap showing Pearson's correlation coefficient between each ChIP-seq replicate of *Pf*ORC1 and *Pf*ORC2. **c)** Genome wide distribution of *Pf*ORC1 and **d)** *Pf*ORC2 sites. **e)** Genome wide distribution of *Pf*ORC$_{1\text{-}2}$ cluster sites. A cluster is defined as a 10 kb region of the genome containing 3 or more *Pf*ORC$_{1\text{-}2}$ sites. **f)** Percentage of GC content of the *Pf*ORC$_{1\text{-}2}$ binding sites. The horizontal bar depicts the GC content of the whole genome (19.3%). **g)** GC content of the genomic regions around the summits of *Pf*ORC binding sites. **h)** Empirical Cumulative Distribution Function (ECDF) of the distances between ChIP peaks of different MACS2 enrichment scores (top 1 %, top 5%, top 10 %, or the bottom 90 %) to the closest G4FS. Randomized datasets are represented as dashed lines. Median distances are indicated. **i)** Heatmap showing the Jaccard statistic of overlap between different *Pf* histone modifications and the *Pf*ORC$_{1\text{-}2}$ peaks. Values range from 0 (no overlap) and 1 (complete overlap). **j)** GC content and *Pf*ORC$_{1\text{-}2}$ density of specific genomic locations. Telomeres defined as the first and last 3kb of each chromosome according to Figueiredo *et al.* 2002. **k)** Enrichment of *Pf*ORC1 and *Pf*ORC2 sites, separately, over scaled coordinates of *var* genes. **l)** Enrichment of *Pf*ORC$_{1\text{-}2}$ sites over scaled coordinates of genes present in heterochromatin genes except for *var* genes.

S3

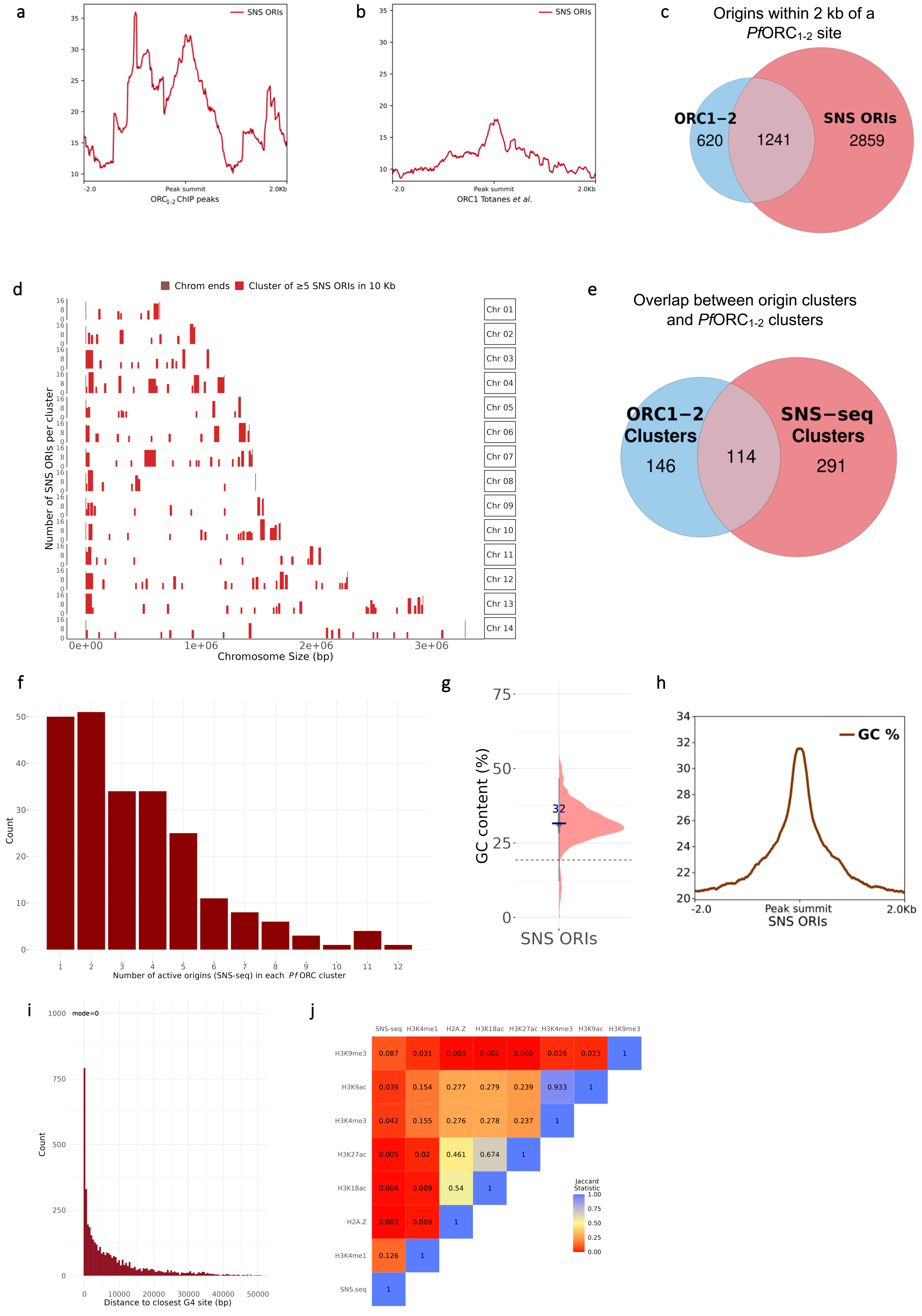
a
SNS ORIs
-2.0
Peak summit
2.0Kb
ORC1-2 ChIP peaks
b
SNS ORIs
ORC1 Totanes et al.
c
Origins within 2 kb of a PfORC1-2 site
ORC1−2
620
1241
SNS ORIs
2859
d
Chrom ends
Cluster of ≥5 SNS ORIs in 10 Kb
Number of SNS ORIs per cluster
Chr 01
Chr 02
Chr 03
Chr 04
Chr 05
Chr 06
Chr 07
Chr 08
Chr 09
Chr 10
Chr 11
Chr 12
Chr 13
Chr 14
0e+00
1e+06
2e+06
3e+06
Chromosome Size (bp)
e
Overlap between origin clusters and PfORC1-2 clusters
ORC1−2 Clusters
146
114
SNS−seq Clusters
291
f
Count
Number of active origins (SNS-seq) in each Pf ORC cluster
g
GC content (%)
32
SNS ORIs
h
GC %
-2.0
Peak summit
2.0Kb
SNS ORIs
i
mode=0
Count
Distance to closest G4 site (bp)
j
SNS-seq
H3K4me1
H2A.Z
H3K18ac
H3K27ac
H3K4me3
H3K9ac
H3K9me3
SNS.seq
Jaccard Statistic
H3K9me3: 0.087 0.031 0.003 0.002 0.002 0.026 0.023 1
H3K9ac: 0.039 0.154 0.277 0.279 0.239 0.933 1
H3K4me3: 0.042 0.155 0.276 0.278 0.237 1
H3K27ac: 0.005 0.02 0.461 0.674 1
H3K18ac: 0.004 0.009 0.54 1
H2A.Z: 0.002 0.009 1
H3K4me1: 0.126 1
SNS.seq: 1

Supplemental figure 3. Genome-wide distribution of SNS-seq origins. a) Enrichment of SNS-seq origins around the peak summit of this study's *Pf*ORC1 peaks and **b)** around the peak summit of the *Pf*ORC1 peaks from Totañes *et al*. 2023 **c)** Number of SNS-seq origins within 2 kb of a *Pf*ORC$_{1-2}$ binding site. **d)** Genome wide distribution of the clusters of SNS-seq origins. **e)** Overlap between SNS-seq clusters (5 or more origins in a 10 kb window) and *Pf*ORC$_{1-2}$ clusters (3 or more *Pf*ORC$_{1-2}$ sites in a 10 kb window). **f)** Number of SNS-seq origins in *Pf*ORC$_{1-2}$ clusters. **g)** Percentage of GC content of the SNS-seq origins. The horizontal bar depicts the GC content of the whole *Pf* genome (19.3%). **h)** GC content of the genomic regions around the summits of SNS-seq origins. **i)** Histogram of the distances between a given SNS-seq origin and the closest G4FS site. **j)** Heatmap showing the Jaccard statistic of overlap between different *Pf* histone modifications and the active origins detected by SNS-seq.

S4

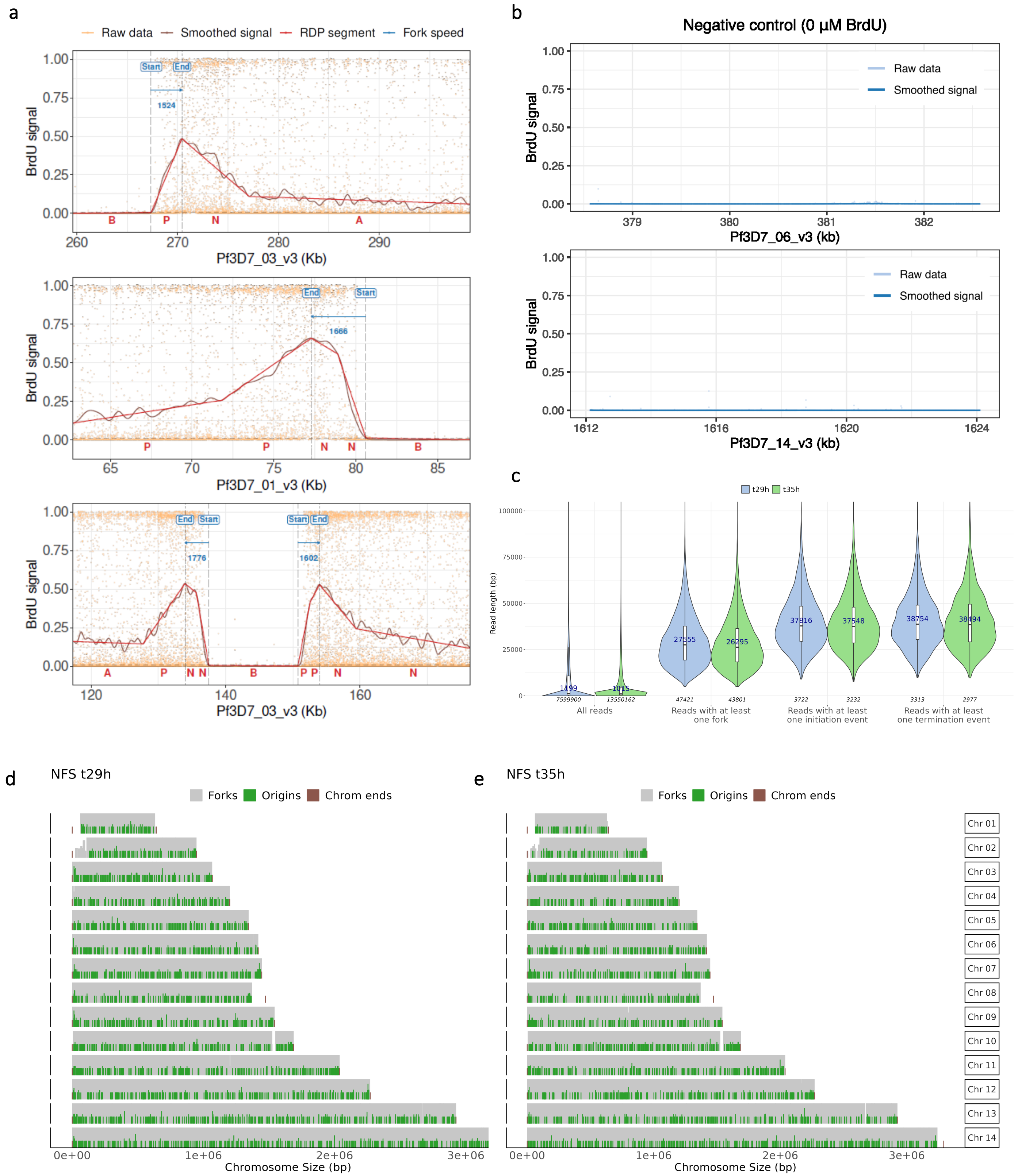

a
Raw data
Smoothed signal
RDP segment
Fork speed
BrdU signal
Start
End
1524
B
P
N
A
Pf3D7_03_v3 (Kb)
1666
Pf3D7_01_v3 (Kb)
1776
1602
b
Negative control (0 µM BrdU)
Pf3D7_06_v3 (kb)
Pf3D7_14_v3 (kb)
c
t29h
t35h
Read length (bp)
1199
1015
27555
26295
37816
37548
38754
38494
7599900
13550162
47421
43801
3722
3232
3313
2977
All reads
Reads with at least one fork
Reads with at least one initiation event
Reads with at least one termination event
d
NFS t29h
Forks
Origins
Chrom ends
Chromosome Size (bp)
e
NFS t35h
Chr 01
Chr 02
Chr 03
Chr 04
Chr 05
Chr 06
Chr 07
Chr 08
Chr 09
Chr 10
Chr 11
Chr 12
Chr 13
Chr 14

Supplemental figure 4. Mapping of active origins and fork speed with NFS and comparison with other mapping datasets. a) Replication fork detection procedure by NFS. **b)** Selected reads from a control nanopore sequencing reaction performed in the absence of BrdU analogue. **c)** Length (bp) of the sequenced reads at t29h and t35h. The number of reads sequenced for each group is indicated in black below the violins and the median read length in blue over the boxplot. **d-e)** Genome wide distribution of replication forks (grey) and initiation events (green) at 29 hpi **(d)** and 35 hpi **(e)**. Different heights indicate enrichment of forks detected in certain genomic regions.

S5

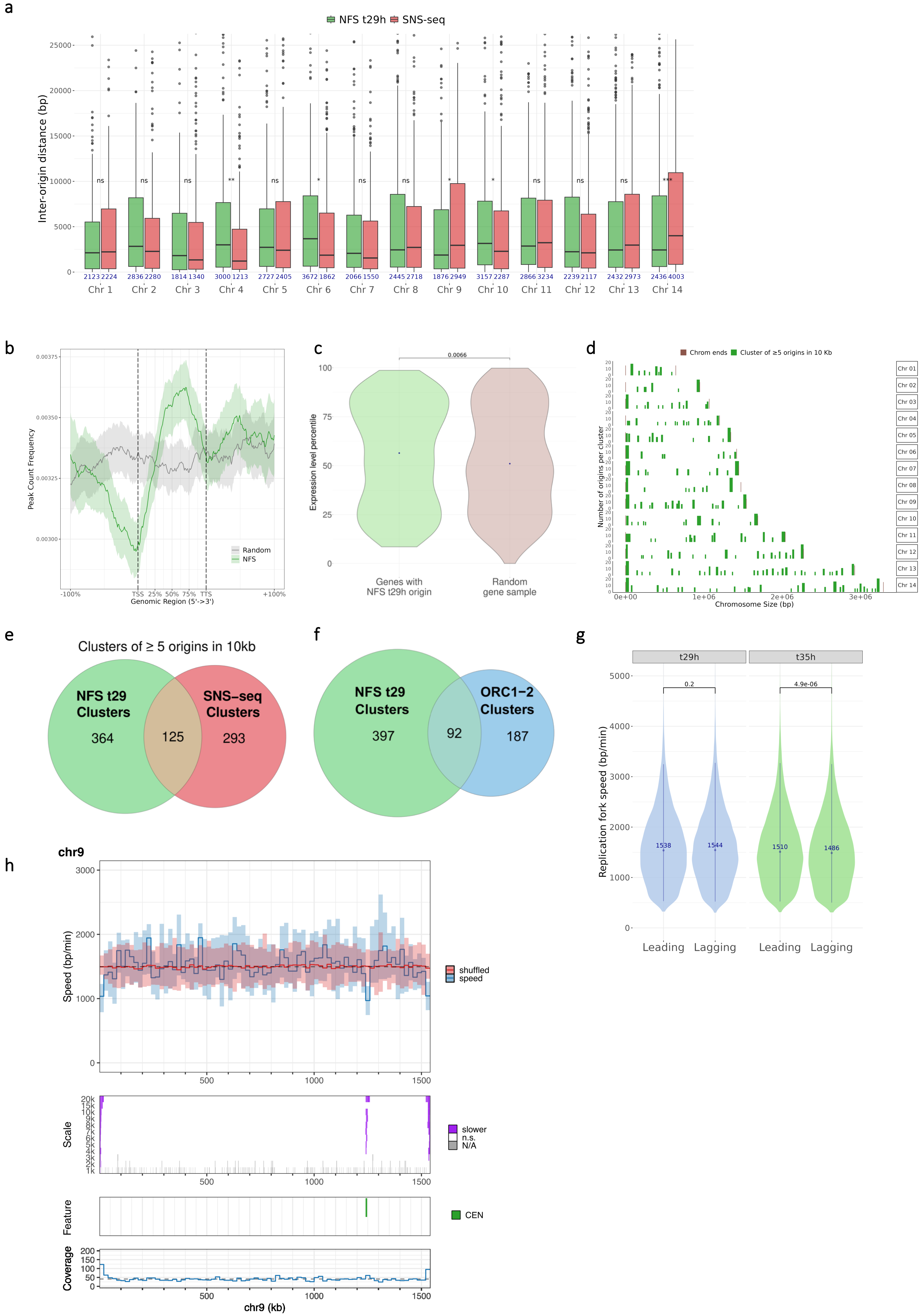
a
NFS t29h
SNS-seq
Inter-origin distance (bp)
25000
20000
15000
10000
5000
0
ns
ns
ns
**
ns
*
ns
ns
*
*
ns
ns
ns
***
2123 2224
2836 2280
1814 1340
3000 1213
2727 2405
3672 1862
2066 1550
2445 2718
1876 2949
3157 2287
2866 3234
2239 2117
2432 2973
2436 4003
Chr 1
Chr 2
Chr 3
Chr 4
Chr 5
Chr 6
Chr 7
Chr 8
Chr 9
Chr 10
Chr 11
Chr 12
Chr 13
Chr 14
b
Peak Count Frequency
0.00375
0.00350
0.00325
0.00300
Random
NFS
-100%
TSS
25%
50%
75%
TTS
+100%
Genomic Region (5'->3')
c
0.0066
Expression level percentile
100
75
50
25
0
Genes with
NFS t29h origin
Random
gene sample
d
Chrom ends
Cluster of ≥5 origins in 10 Kb
Number of origins per cluster
Chr 01
Chr 02
Chr 03
Chr 04
Chr 05
Chr 06
Chr 07
Chr 08
Chr 09
Chr 10
Chr 11
Chr 12
Chr 13
Chr 14
0e+00
1e+06
2e+06
3e+06
Chromosome Size (bp)
e
Clusters of ≥ 5 origins in 10kb
NFS t29
Clusters
364
125
SNS–seq
Clusters
293
f
NFS t29
Clusters
397
92
ORC1–2
Clusters
187
g
t29h
t35h
Replication fork speed (bp/min)
5000
4000
3000
2000
1000
0
0.2
4.9e-06
1538
1544
1510
1486
Leading
Lagging
Leading
Lagging
h
chr9
Speed (bp/min)
3000
2000
1000
0
500
1000
1500
shuffled
speed
Scale
20k
15k
10k
9k
8k
7k
6k
5k
4k
3k
2k
1k
slower
n.s.
N/A
Feature
CEN
Coverage
200
150
100
50
0
chr9 (kb)

Supplemental figure 5. Comparison of NFS mapping with other mapping datasets and fork speed analysis. **a)** Distribution of inter-origin distances measured by SNS-seq or NFS at 29hpi for each chromosome. Median distances are shown in blue as well as the results of two-sided Wilcoxon tests (ns: $p > 0.05$, *: $p <= 0.05$, **: $p <= 0.01$, ***: $p <= 0.001$, ****: $p <= 0.0001$). **b)** Enrichment of NFS origins (green) over scaled coordinates of *P. falciparum* genes. A randomized (control) dataset in shown in grey. TSS corresponds to the transcription Start Site and TTS to the transcription termination site of each gene. Regions of equal length to each gene are included upstream and downstream the TSS and TTS, respectively. **c)** Violin plots depicting the expression percentile of the genes displaying an enrichment of t29h NFS origins and a randomized dataset of equal number in brown. The result of a two-sided Wilcoxon test is shown. Expression percentiles were obtained from Chappell et al. 2020. **d)** Genome wide distribution of t29h NFS origin clusters. A cluster is defined as a 10 kb region containing 5 or more NFS origins. **e)** Overlap between NFS and SNS clusters. **f)** Overlap between NFS and *Pf*$ORC_{1\text{-}2}$ clusters. **g)** Replication fork speed on leading and lagging strands at 29 hpi and 35 hpi. Median fork speeds are indicated above the blue point. **h)** Example of NFS Speed map for chromosome 9. Although fork speed decreases towards the ends of the chromosome and over the centromere, this is not due to a depletion in coverage reads. Chromosome 9 is shown. Panels from top to bottom: (1) median of experimental fork speeds (blue line) with 98% confidence interval of the median (light blue) and median of reshuffled speeds (red line) with 98% confidence interval of the median (light red) computed in 20 kb windows (dotted line, median fork speed in the whole genome); (2) results of Mann–Whitney-Wilcoxon tests with Holm correction (one-sided) performed along the chromosome to compare the speed distribution in a given window of a given width (1, 2, 3, 4, 5, 6, 7, 8, 9, 10, 15 and 20 kb) to the speed distribution on the whole genome (purple, regions of lower fork speed; white, n.s., not significant; statistical significance was set to $p < 0.01$; grey, N/A, not applicable, regions with no forks); (3) position of selected genomic features (CEN, centromere); (4) coverage of individual replication fork velocities (dotted line, median coverage of the genome).
